# Terracing influences soil microbial assembly in citrus orchards: stochastic processes dominate community dynamics in a karst sloping land

**DOI:** 10.1186/s12866-026-04811-4

**Published:** 2026-02-25

**Authors:** Jiaojiao Zhang, Yuxin Dai, Adnan Mustafa, Liwen Li, Yuxuan Li, Tongfang Sun, Minglei Chen, Hao Yang, Jiangming Ma

**Affiliations:** 1 Key Laboratory of Ecology of Rare and Endangered Species and Environmental Protection (Guangxi Normal University), Ministry of Education/Guangxi Key Laboratory of Landscape Resources Conservation and Sustainable Utilization in Lijiang River Basin, Guilin, 541006 China; 2 College of Life Science, Guangxi Normal University, Guilin, 541006 China; 3 Guangdong Provincial Key Laboratory of Applied Botany, South China Botanical Garden, Chinese Academy of Sciences, Guangzhou, 510650 China

**Keywords:** Soil microbial assembly, Stochastic processes, Soil metabolites, Citrus orchards, Karst sloping land, Terraces practice, Nitrogen cycling

## Abstract

**Background:**

Terracing is a key soil conservation practice in karst citrus orchards, yet its long-term effects on rhizosphere microbial community assembly remain poorly understood, especially the relative influence of deterministic (e.g., environmental filtering) versus stochastic processes (e.g., dispersal limitation).

**Results:**

We investigated rhizosphere soil microbial communities along a terrace chronosequence (0–12 years) in the Lijiang River Basin using MiSeq sequencing and metabolomics, with null model analysis employed to assess community assembly processes. Terrace age did not significantly affect microbial α-diversity, but was associated with subtle changes in community composition: Proteobacteria, a copiotrophic group, decreased slightly, while Chloroflexi, an oligotrophic group, increased modestly. These shifts suggest a weak trend toward lower soil nutrient availability rather than a clear successional reorganization. Microbial diversity and structure were significantly correlated with soil stoichiometric ratios and available phosphorus (*p* < 0.05). Terracing also affected microbial network complexity and potential function. Potential functional profiling and metabolome data revealed that L-glutamine, a key nitrogen source, was negatively correlated with potential catabolic nitrate reduction (*p* < 0.05). This relationship was most pronounced at the Y5 phase (peak diversity/network complexity), suggesting accelerated L-glutamine utilization tightly coupled with enhanced potential for dissimilatory nitrate reduction to maximize nitrogen-use efficiency during the successional climax. Notably, stochastic processes explained over 96% of the microbial assembly. Bacterial communities were primarily driven by homogenizing dispersal, while fungal communities followed undominated processes.

**Conclusion:**

The prominence of stochasticity in our results complements current understanding of agricultural microbiome assembly, particularly emphasizing its vital role in fragile karst environments. We propose that optimizing terrace rotation intervals (e.g., every 5-year) could be a practical strategy to enhance nitrogen-cycling efficiency and support sustainable nutrient management in karst citrus cultivation.

**Graphical Abstract:**

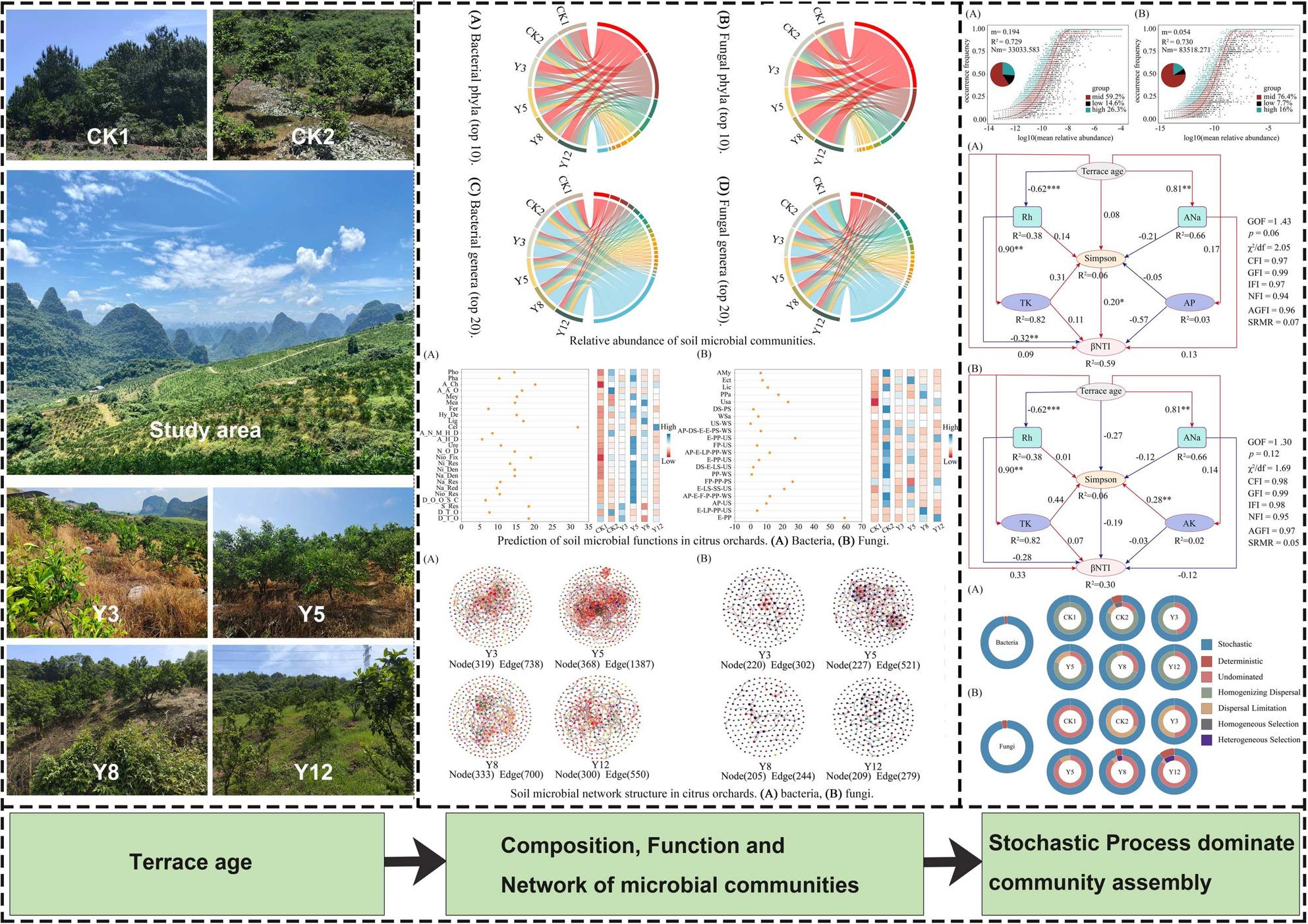

**Supplementary Information:**

The online version contains supplementary material available at 10.1186/s12866-026-04811-4.

## Introduction

Karst landscapes, characterized by their unique geological formations and fragile soil structures, are particularly vulnerable to land degradation and nutrient loss, making them globally recognized as critical zones for ecological conservation and sustainable development [1, 2]. In these regions, agricultural development faces significant challenges due to severe soil erosion, rocky desertification, and limited arable land driven by steep slopes, shallow soils, and historically unsustainable land-use practices [3, 4]. Addressing these constraints is essential for achieving food security and ecological stability, particularly in rural areas where agriculture underpins poverty alleviation and economic revitalization [3]. This is particularly true for regions, such as southwestern China, where citrus cultivation is a key agricultural activity and the management of sloping karst land presents both ecological and agronomic challenges.

Terracing has emerged as a transformative strategy for reconciling agricultural productivity with environmental protection in mountainous landscapes. By converting slopes into stepped platforms, terracing mitigates soil erosion, enhances water retention, and improves soil fertility through increased organic carbon and nutrient accumulation [5]. These modifications not only stabilize soil structure but also promote vegetation recovery in arid zones and enhance carbon sequestration [5]. Moreover, terracing exerts a positive influence on mycorrhizal colonization and effectively controls spore loss through surface runoff entrainment [6]. Furthermore, terracing drives a shift in the microbial community from oligotrophic to copiotrophic guilds, and the conserved SOC supplies ample energy, enabling microbes to invest more resources in growth and competitiveness, thereby sustaining a stable population [7]. However, although terracing has been shown to markedly reshape soil physicochemical properties and microbial communities, the ecological consequences of terracing, especially its long-term effects on soil microbial communities remain poorly understood.

Karst terraces create fragmented microhabitats due to bedrock fractures and heterogeneous soil accumulation. The structure of microbial communities is shaped by a combination of deterministic and stochastic ecological processes, and understanding community assembly is crucial for describing these influences [8]. While deterministic processes dominate in stable environments, stochastic processes prevail in heterogeneous or disturbed systems [9]. Vellend [10] categorized the processes governing community assembly into four main mechanisms: selection, dispersal, speciation, and drift. Quantifying the relative contributions of deterministic (e.g., selection) and stochastic (e.g., drift and dispersal) processes has since become a central focus in microbial ecology. Numerous studies have employed quantitative approaches to assess microbial community [11]. Among them, Stegen’s approach has been widely used due to its robustness in distinguishing between deterministic and stochastic influences on microbial community assembly across diverse ecosystems [11]. This method enables the precise quantification of ecological assembly mechanisms, offering critical insights into how microbial communities respond to environmental gradients and land-use changes [9]. Deterministic microbial assembly, primarily driven by environmental filtering, is a prevailing feature in agricultural systems globally [12]. This pattern is well-documented across diverse regions, including paddy fields in subtropical China [13], wheat–maize rotations on the North China Plain [14, 15], and cultivated soils across the continental United States [16]. However, in more heterogeneous or transitional landscapes, such as terraced karst slopes, where soil formation, hydrology, and plant–microbe interactions vary over space and time, the ecological and environmental sensitivity remains high, vulnerability is strong, and stability is low, the relative influence of stochastic processes may be considerably greater [17]. Testing this dichotomy is critical for karst-specific conservation strategies. Notably, such study differs distinctly from general agricultural soil microbial assembly research, and this distinction is closely tied to the unique environmental characteristics of karst regions. Unlike the relatively homogeneous soil conditions, moderate calcium levels, and sufficient phosphorus supply in most conventional agricultural soils, karst environments are defined by high calcium, low phosphorus, and extreme habitat heterogeneity, which collectively shape microbial communities in specific ways. The relative contribution of microbial community assembly is influenced by multiple factors. Hence, understanding the assembly mechanisms of soil microbial communities and influencing factors, such as soil pH, organic matter, total phosphorus, and anthropogenic environmental changes, is crucial for predicting ecosystem functions and designing sustainable land management strategies [18].

The rhizosphere microbial community plays a pivotal role in mediating plant nutrient acquisition, stress tolerance, and productivity. In perennial orchard systems like citrus, long-term land management practices (e.g., terracing age) may progressively modify rhizosphere conditions, which in turn may alter microbial community composition and ecosystem functioning. Many studies have revealed the assembly patterns and driving factors of microbial communities in grassland [19], as well as agricultural [20] and river ecosystems [21]. However, in karst terraces—a system defined by high calcium content, acidic soils, and fragmented microhabitats—the interplay between terrace age and microbial assembly mechanisms remains unresolved. Specifically, it is unclear whether microbial communities shift toward deterministic selection as terraces stabilize or whether dispersal limitation maintains stochastic dominance. Addressing these issues is critical for designing microbiome-informed strategies to enhance agricultural sustainability. Therefore, we hypothesized that long-term terracing induces progressive nutrient limitations, creating selective pressures that favor oligotrophic microbial taxa. This environmental filtering may subsequently shift community assembly dynamics, with fungal communities particularly demonstrating susceptibility to deterministic processes due to their distinct life history strategies, including mycelial growth strategy and stronger niche specificity during organic matter decomposition [22].

Citrus fruits are widely consumed worldwide and are of high economic importance [23]. The Lijiang Karst River Basin has a long history of citrus cultivation and a vast area of cultivation. The Lijiang areas face escalating soil degradation due to intensive cultivation on steep slopes. Local farmers increasingly adopt terracing to curb erosion and boost yields, yet the ecological consequences of long-term terrace use—particularly for microbial diversity and function—are unknown. To address this gap, we integrated high-throughput sequencing and null model analysis to investigate: (1) how bacterial and fungal communities diverge across terrace chronosequences, (2) the soil properties that drive these shifts, and (3) whether stochastic or deterministic processes govern microbial assembly in karst terraces. By resolving these issues, our study provides actionable insights for optimizing terrace management, enhancing nutrient cycling, and promoting sustainable citrus cultivation in ecologically fragile karst landscapes.

## Materials and methods

### Study area

**Fig. 1:**
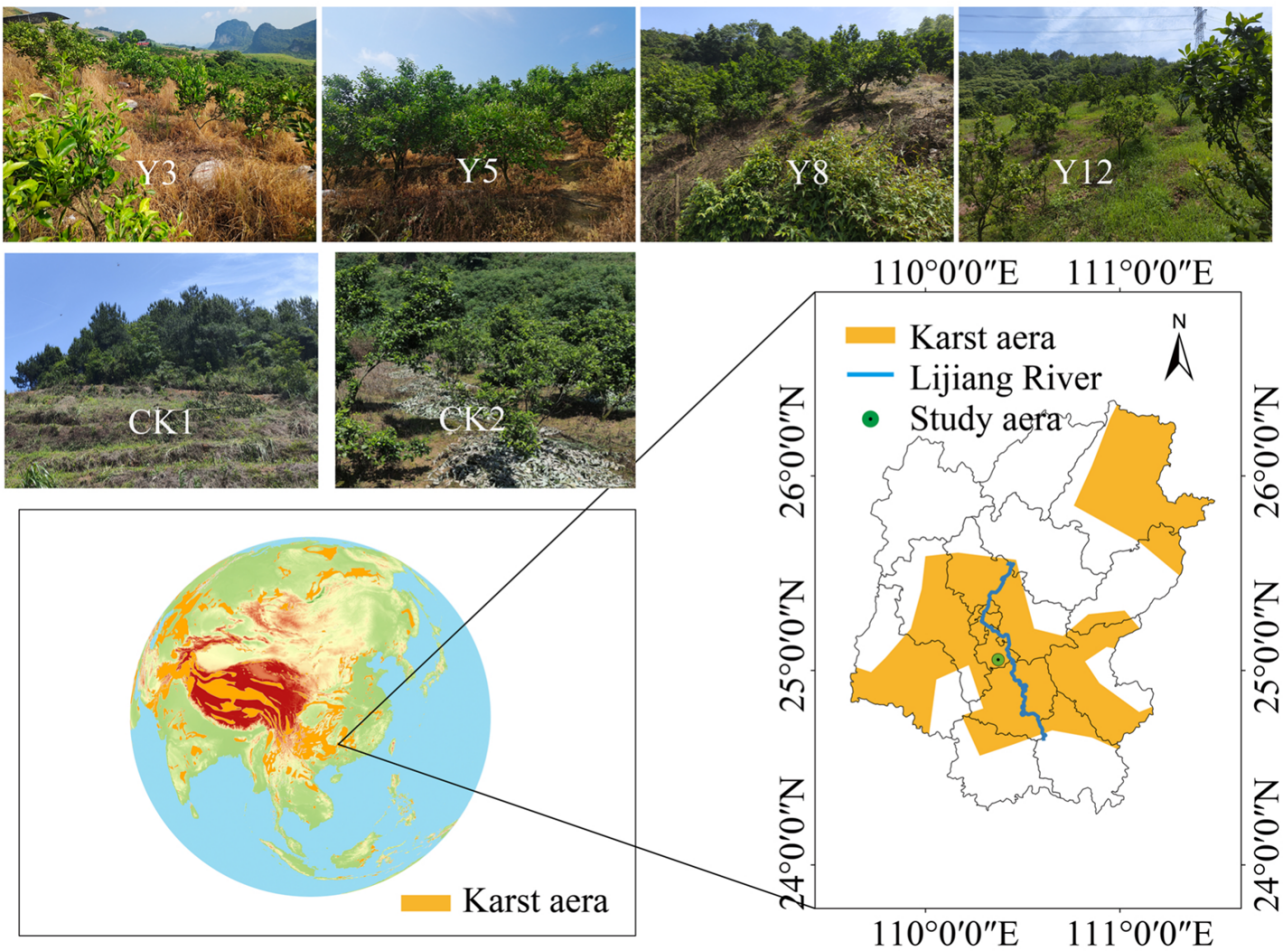
Location of sampling sites. The terraced citrus orchards for three year (Y3), five year (Y5), eight year (Y8), twelve year (Y12), Natural forests (CK1) and the non-terraced sloped farmland (CK2)

The study area was conducted in the Lijiang River Basin (24°18′–25°41′N and 109°45′–110°40′E), a UNESCO World Heritage Site in South China (Fig. 1). The region is characterized by shallow soils overlying extensive carbonate bedrock, forming a representative karst landscape [24]. According to the Chinese Soil Classification Taxonomy, the dominant soil types are lateritic red soils and loess-derived calcareous soils [25]. Soil physicochemical properties for the experimental plots are comprehensively presented in Table S1 (see Supplementary Materials). The area experiences a subtropical monsoon climate with distinct seasonal variation. Annual precipitation averages approximately 2,200 mm with the majority occurring during the summer months (June–August). Mean temperature ranges from 11.5 °C in January to 28.1 °C in July [26]. These climatic and edaphic conditions, coupled with the region’s fragile topography, make it highly sensitive to soil degradation and nutrient loss, particularly under intensive land-use practices.

### Experimental design and layout description

Soil sampling was implemented in July 2022 (peak hot-rainy season) to capture critical biogeochemical processes in karst ecosystems governed by carbonate bedrock hydrogeology. This annual sampling window (July–August) coincides with maximum monthly precipitation (300 mm) and mean temperature (28.1 °C), which collectively stimulate microbial processes through multiple mechanisms. Suitable temperatures and rainfall promote the secretion of plant root exudates and nutrient accumulation, enhancing root nutrient content and thereby benefiting microbial activity and growth [26]. Due to seasonal differences in temperature and humidity, soil microbial communities exhibit distinct temporal dynamics: although microorganisms break dormancy in March, their enzyme activity remains low; in May and July, elevated enzyme activity coincides with high microbial functional demand and adaptive responses to nutrient fluctuations, and in September, ecological conditions stabilize, but microbial interactions weaken and spatial distribution becomes more scattered [26]. Specifically, the July sampling targeted three key microbial stimulation mechanisms: (1) elevated temperatures, which optimize enzymatic catalysis rates (e.g., cellulase, phosphatase) for organic matter decomposition [26]; (2) saturated soil conditions, which mitigate karst-specific hydraulic limitations, facilitating microbial dispersal and biofilm formation [26]; and (3) fruit-expansion phase of citrus trees, ensuring stabilized rhizodeposition (e.g., organic acids, soluble sugars) to minimize phenology-driven fluctuations in microbial community composition [27]. Agricultural management practices (fertilization, irrigation, and tillage) were standardized across all study sites to isolate temporal effects from transient nutrient inputs. Standardized nitrogen fertilization (179 kg N·ha⁻^1^·yr⁻^1^) was applied across all terraces from December 2021 to February 2022 [28]. Five-month soil stabilization post-fertilization ensured complete nitrification–denitrification equilibration and C:N:P stoichiometric stabilization, effectively eliminating transient nutrient artifacts [28]. Surface soil horizons (0–20 cm depth) were systematically sampled given their biogeochemical primacy in karstic citrus systems: the fiber root density in this layer is high, and the carbon input in the citrus rhizosphere is partially balanced [29]. Stratified analysis of microbial communities confirmed that the activation of β-glucosidase activity and phosphorus phosphatase activity occurred in this layer, reflecting the limitations of the evolution of shallow soil in karst landforms [30].

### Soil samples collection

The soil samples were collected from a representative citrus plantation in Lijiang River Basin comprising terraces with a slope of approximately 20°. Stratification was performed based on citrus orchard age for three, five, eight, and twelve years (i.e., 2019, 2017, 2014, and 2010), designated as Y3 (3 years), Y5 (5 years), Y8 (8 years), and Y12 (12 years), respectively, to evaluate orchards representing different terraced cultivation transition periods. Control sites were established in natural forest (CK1) and uncultivated slopes (CK2). CK1 served as an undisturbed reference ecosystem to establish microbial community baseline conditions, while CK2 represented pre-terracing soil conditions, functioning as a non-intervention control for citrus cultivation soils to eliminate the influence of citrus rhizosphere on soil microbial communities. The maximum citrus plant age of 12 years was selected because fruit quality tends to decline after a decade, after which the trees are typically removed and replanted.

Following farmland soil environment-quality monitoring technology standards (NY/T 395–2012), 144 soil samples were collected and split for physicochemical, microbial, and metabolite analyses. According to the soil background conditions, 20 × 20 m plots were established on each slope, and 5 sub-plots were selected according to the five-point sampling method. In each subplot, target citrus trees were selected, litter and impurities around the target citrus trees were removed, and 0–20 cm soil layer samples were collected vertically around the target citrus trees using stainless steel soil drill [28]. After removing gravel and roots, the samples were mixed with equal quality, placed in polyethylene plastic bags, labeled, transported at 4 °C within 24 h, and refrigerated to ensure microbial activity [28].

### Determination of soil physico-chemical properties

The soil water content (Rh) was measured gravimetrically by oven-drying at 105 °C for 12 h [31]. Soil pH was determined using a calibrated pH meter (Mettler Toledo, Switzerland) in a 1:2.5 (w/v) soil-deionized water suspension [32]. Total carbon (TC) and total nitrogen (TN) were quantified via dry combustion using an Elementar Vario MAX CNS analyzer (Elementar Analysensysteme GmbH, Germany) [33]. Soil organic carbon (SOC) was assessed via the potassium dichromate oxidation method, with organic matter (OM) calculated using the Van Bemmelen conversion factor (1.72) [34]. Available calcium (ACa), magnesium (AMg), and sodium (ANa) were extracted with 1 M ammonium acetate (pH 7.0) and quantified by inductively coupled plasma mass spectrometry (ICP-MS; Thermo Fisher iCAP R) [35]. Total phosphorus (TP) and available phosphorus (AP) were measured using the molybdenum blue colorimetric method [36], calculated based on the 3σ blank standard deviation, with a minimum detectable limit (MDL) of 0.01 mg/L. Available potassium (AK) was determined by flame photometry (Jenway PFP7, UK) following ammonium acetate extraction, while total potassium (TK) was analyzed via flame photometry after HNO₃–HClO₄ digestion [37], with a detection limit of 0.01–0.6 mg/L (signal-to-noise ratio ≥ 3). Soil texture was classified by the hydrometer method [38]. The statistical results of soil physicochemical properties are listed in Table S1.

### Soil DNA extraction, PCR, and high-throughput sequencing

Genomic DNA was extracted from 0.5 g of rhizosphere soil samples using the MoBio Power Soil DNA extraction kit (MoBio Laboratories, Inc., CA, USA) following the manufacturer’s instructions and stored at − 80 °C until PCR amplification. Quawell Q5000 (Quawell Technology, Inc., San Jose, USA) was utilized to determine the concentration and purity of the extracted DNA. To amplify bacterial 16S rRNA and fungal internal transcribed spacer (ITS) rRNA, specific primers 341 F (5'-CCTACGGGNGGCWGCAG-3') and 805R (5'-GACTACHVGGGTATCTAATCC-3') were used for PCR amplification targeting the V3-V4 hypervariable regions of 16S rRNA; for fungi, the primers ITS1F (5′‑CTTGGTCATTTAGAGGAAGTAA‑3′) and ITS2R (5′‑GCTGCGTTCTTCATCGATGC‑3′) were used for PCR amplification of the ITS region of ITS rRNA. The PCR reaction products were evaluated by electrophoresis on a 2% agarose gel, pooled and purified using the QIAquick PCR purification kit (Qiagen, Valencia, CA, USA), and quantified with the NanoDrop ND-2000 (Thermo Scientific, Waltham, MA, USA). Purified PCR products from all samples were mixed in equi molar amounts, and Illumina MiSeq ultra-high-throughput sequencing was performed by Sangon Biotech Co., Ltd. (Shanghai, China) [28]. Sequences were processed using the QIIME 1.9.1 pipeline. After quality filtering and chimera removal, high-quality sequences were clustered into operational taxonomic units (OTUs) at a 97% similarity threshold. Taxonomic assignment was performed with a confidence threshold of 0.8 using the SILVA database for bacterial 16S rRNA gene OTUs and the UNITE database for fungal ITS OTUs.

### Soil metabolite extraction and liquid chromatography-tandem mass spectrometry (UHPLC-MS/MS) analysis

We addressed the inherent limitations of taxonomic-based functional predictions (FAPROTAX and FUNGuild) by cross-validating the resulting microbial profiles against targeted metabolomics data. Key nutrient metabolites, such as L-glutamine, served as empirical proxies to confirm the predicted in situ biochemical activities.

Soil metabolites were extracted using a solvent mixture of methanol, acetonitrile, and water (2:2:1, v/v/v). The mixture was vortexed for 1 min, followed by 30 min of low-temperature ultrasonication to maximize extraction yield. After incubating at − 20 ℃ for 30 min, samples were centrifuged at 13,000 × g for 15 min at 4℃. The supernatant was then transferred to injection vials for analysis. Each treatment was repeated four times, and quality control (QC) samples were prepared by pooling 20 μL of supernatant from each sample, with the remaining volume used for metabolomics analysis [39].

Metabolite analysis was performed using UHPLC-MS/MS. The system comprised a Vanquish UHPLC (Thermo Fisher, Germany) coupled to an Orbitrap QE HF-X mass spectrometer (Thermo Fisher, Germany) [40]. Chromatographic separation was achieved on an ACQUITY UPLC HSS T3 column (100 × 2.1 mm ID, 1.8 μm; Waters). The mobile phase consisted of solvent A (95% water–5% acetonitrile with 0.1% formic acid) and solvent B (47.5% acetonitrile–47.5% isopropanol–5% water with 0.1% formic acid) [41]. MS signals were acquired using electrospray ionization in alternating positive and negative ion scanning modes. QC samples were run at every 5–15 analytical samples to monitor and ensure analytical reliability. Raw data processing was conducted using Progenesis QI (Waters) for peak alignment, identification, retention time correction, and baseline filtering. This generated a data matrix containing peak intensity, mass-to-charge ratio, and retention time. Metabolite identification relied on aligning MS2 spectra against multiple databases, including KEGG, HMDB, Metabolite Link, and Majorbio, maintaining a mass error threshold below 10 ppm [40]. All metabolomics analyses were performed by Majorbio Biotechnology Co., Ltd.

### Statistical analyses

Data processing and preliminary analyses were carried out using Microsoft Excel 2019 (Microsoft Corp., WA, USA) and SPSS (version 27.0; IBM, Chicago, IL, USA). Advanced statistical analyses and visualization were performed with Origin Pro 2024 (Origin Lab Corporation, Northampton, MA, USA) and the R software (version 4.4.3 R Core Team, 2024). For group comparisons, Student’s t-test was used for two groups and one-way ANOVA for multiple groups. Prior to these analyses, data normality was verified through Shapiro–Wilk tests with a significance level set at *p* > 0.05. The Spearman correlation coefficient was applied to assess relationships between variables with non-normal distributions, while Pearson rank correlation coefficient was used for variables following a normal distributions.

Microbial diversity was evaluated using several metrics: the Sobs index for community composition, Chao1 and ACE estimator for community abundance, and the Shannon index for community diversity. Correlation heatmaps were generated using the “pheatmap” package in R, and *p*-values were adjusted using the Benjamini–Hochberg method. Mantel tests were computed and visualized with the “linkET” and “ggplot2” packages in R, with *p*-values adjusted by the false discovery rate (FDR). Redundancy analysis (RDA) was used to quantify and visualize the associations between soil microbial community diversity and soil physicochemical properties. Significance was determined through 999 permutations (*p* < 0.05), and the analysis was performed using the “vegan” package in R software (version 4.4.3; R Core Team, 2024) [42]. Co-occurrence networks were constructed in R (version 4.4.3) using the “Hmisc” and “igraph” packages with SparCC (Sparse Correlations for Compositional Data); Spearman correlation coefficient (*p*) was calculated, and the network was filtered based on coefficient thresholds of *r* > 0.8 and *p* < 0.001. Network topology properties (e.g., average degree, clustering coefficient, and modularity) were computed using the “igraph” package. Node roles in the network were classified based on within-module connectivity (Zi) and among-module connectivity (Pi): hubs (Zi > 2.5, Pi < 0.62), connectors (Zi < 2.5, Pi > 0.62), module hubs (Zi > 2.5, Pi > 0.62), and peripherals (Zi < 2.5, Pi < 0.62). Keystone taxa were identified as hubs or connectors with strong topological importance, characterized by high degree and betweenness centrality. Network topology was visualized in Gephi (version 0.9.2). Functional profiling of microbial communities was performed via the Sangon Biotechnology Cloud Platform (https://ngs.sangon.com/). Bacterial functional profiles were predicted using the FAPROTAX database, which annotates ecological functions such as carbon and nitrogen cycling based on bacterial taxonomic information. Fungal functional guilds were predicted with the FUNGuild database using the taxonomic annotation of fungal OTUs. To ensure the accuracy and reliability of functional annotation, only predictions with confidence levels of “Highly Probable” or “Probable” were retained for downstream ecological statistical analysis. Predictions with a confidence level of “Possible” were excluded to prevent overgeneralization of functional guilds caused by low-confidence annotations. The study area was mapped using ArcGIS software (version 10.8).

Neutral community models (NCMs) were fitted to disentangle ecological assembly mechanisms and assess stochastic processes. Model fit (R^2^) and migration rate (Nm) were calculated, where higher R^2^ values (greater than 0.7) indicated stronger stochastic influences. Phylogenetic turnover was evaluated using β-nearest taxon index (βNTI) via the picante package in R (R Core Team, 2024). Communities with |βNTI|> 2 were classified as deterministically structured, with βNTI < − 2 indicating homogeneous selection and βNTI > 2 indicating variable selection. Communities with |βNTI|< 2 implied stochastic dominance.

Structural equation modeling (SEM) was employed to assess the direct and indirect effects of terrace age on soil microbial community assembly. Path fitting and parameter estimation were performed using the “lavaan” package in R with the maximum likelihood method. The overall goodness-of-fit of the model was evaluated against several criteria: chi-square test (χ^2^, *p* > 0.05), comparative fit index (CFI > 0.90), Tucker–Lewis index (TLI > 0.90), root mean square error of approximation (RMSEA < 0.05), and standardized root mean square residual (SRMR < 0.08).

## Results

### Microbial community variation across terrace chronosequence

**Fig. 2:**
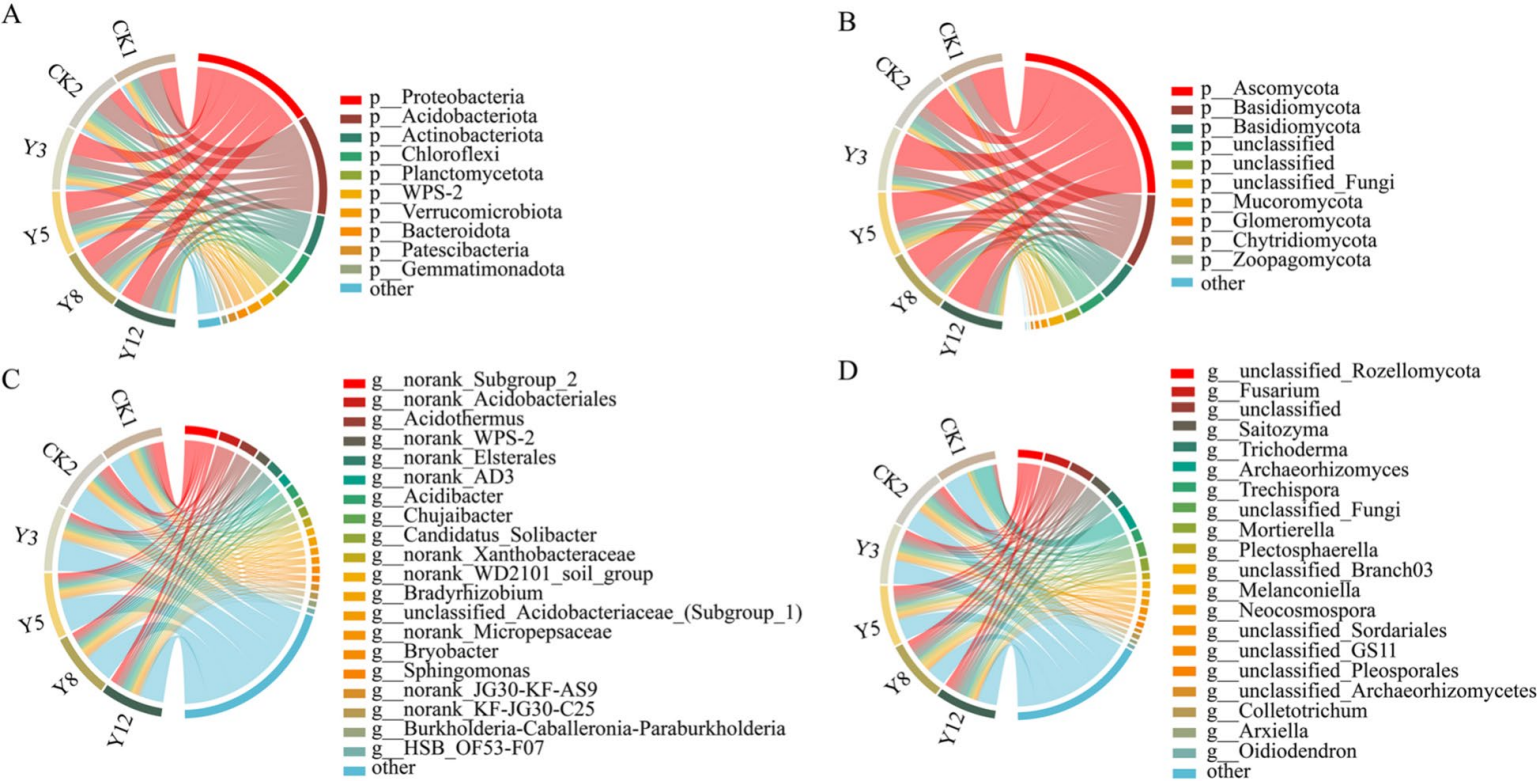
Relative abundance of soil microbial communities. **A** Bacterial phyla (top 10). **B** Fungal phyla (top 10). **C** Bacterial genera (top 20). **D** Fungal genera (top 20). CK1: Natural forest; CK2: the non-terraced sloped farmland; Y3-Y12: terraces aged 3–12 years

Figure 2 illustrates the relative abundance of dominant bacterial and fungal communities at the phylum level (top 10 phyla), along with the top 20 genera for each domain. Among bacteria, Proteobacteria (22.50–37.10%), Acidobacteria (19.97–32.50%), and Actinobacteria (6.98–14.84%) were the most prevalent bacterial phyla, collectively accounting for 61.99–76.32% of total bacterial sequences across all treatments (Fig. 2A). Fungal communities were dominated by Ascomycota (46.81–57.84%), Basidiomycota (13.66–26.76%), and Rozellomycota (2.07–15.98%), representing 75.95–86.75% of fungal sequences (Fig. 2B). The age of terraced citrus orchards drove microbial community variation. Both Proteobacteria and Ascomycota relative abundances were higher in terraced citrus orchards of different ages than in CK2, with Proteobacteria relative abundance exceeding 1.53 times that of CK2. Conversely, the relative abundance of Chloroflexi was lower than that in CK2 but increased with citrus orchard age.

Notably, Proteobacteria abundance decreased by 2.74% from 3- to 12-year terraces, while Chloroflexi increased by 4.72%. Acidobacteria reached peak abundance (22.26%) in 5-year terraces (Y5), coinciding with the lowest Actinobacteria (8.26%) and Ascomycota (52.45%) levels. At the genus level, *norank_Subgroup_2* (Acidobacteria), *norank_Acidobacteriales*, and *Acidothermus* dominated bacterial communities (Fig. 2C), whereas *unclassified_Rozellomycota*, *Fusarium*, and *Saitozyma* prevailed among fungi (Fig. 2D).

Bacterial ACE, Chao1, and Sobs indices showed significant quadratic responses to terraced citrus orchards (*p* < 0.05, ANOVA). ACE and Chao1 indices peaked in Y5 before declining in older systems (Y12) (Fig. S1A, B, D). In contrast, fungal communities exhibited divergent trends: Shannon and Sobs indices remained stable (*p* > 0.05), while ACE and Chao1 indices were the highest in CK1 and Y5 (Fig. S1E, F, G, H). Strikingly, bacterial ACE, Chao1, and Sobs indices (α-diversity) in CK1 were 8.74–24.09%, 8.38–23.26%, and 11.5–27.78% lower than those in terraced systems (*p* < 0.05), underscoring terracing’s role in enhancing microbial complexity. The Sobs index for bacteria peaked in CK2, whereas fungal Sobs maxima occurred in CK1 (Fig. S1D, H), reflecting distinct legacy effects of land-use history on microbial richness.

### Functional dynamics of microbial communities across terrace ages

FAPROTAX analysis revealed potential metabolic specialization linked to terrace age (Fig. S2A). Aerobic chemoheterotrophy dominated microbial potential functions, peaking in Y12, followed by Y5. The potential for nitrogen-cycling functional capabilities, including nitrogen fixation and nitrate reduction, initially increased but declined after 8 years, though the potential for nitrogen fixation persisted (*p* < 0.05). Cellulolytic potential capabilities emerged as the core carbon-related function, while the potential for hydrocarbon degradation surged in older terraces. Conversely, potential sulfur-oxidizing capabilities declined with terraced citrus orchard age, aligning with reduced soil C/P, suggesting a potential metabolic shift toward nitrogen utilization. These results indicate that the Y5 stage represents a transitional period where key potential nitrogen-cycling functions remain prominent alongside emerging potential carbon-degradation capabilities, highlighting its distinct functional profile in terrace succession.

The functional attributes of fungal communities were predicted using FUNGuild. Based on predictions with “Highly Probable” or “Probable” confidence levels, our results highlighted fungal trophic flexibility, with multiple potential trophic modes, including Pathotroph, Saprotroph, Symbiotroph, and transitional guilds found in the soil fungal community, among which transitional trophic guilds were predicted to be dominant (Fig. S2B). In CK2, potential multi-functional fungi (e.g., animal pathogen-endophyte-fungal parasite) were abundant. Y3 was associated with a higher potential abundance of favored animal pathogen-dung saprotroph-endophyte-epiphyte-plant saprotroph-wood saprotroph guilds, while Y5 exhibited thriving ectomycorrhizal and endophyte-plant pathogen-undefined saprotroph guilds. Meanwhile, the relative potential abundance of functional guilds, such as endophyte-lichen parasite-plant pathogen-undefined saprotroph, plant pathogen in the 8-year terrace, was relatively high. At 12 years, the potential dominance of endophyte-plant pathogen dominance indicated a potential stabilization mutualistic interactions.

**Fig. 3:**
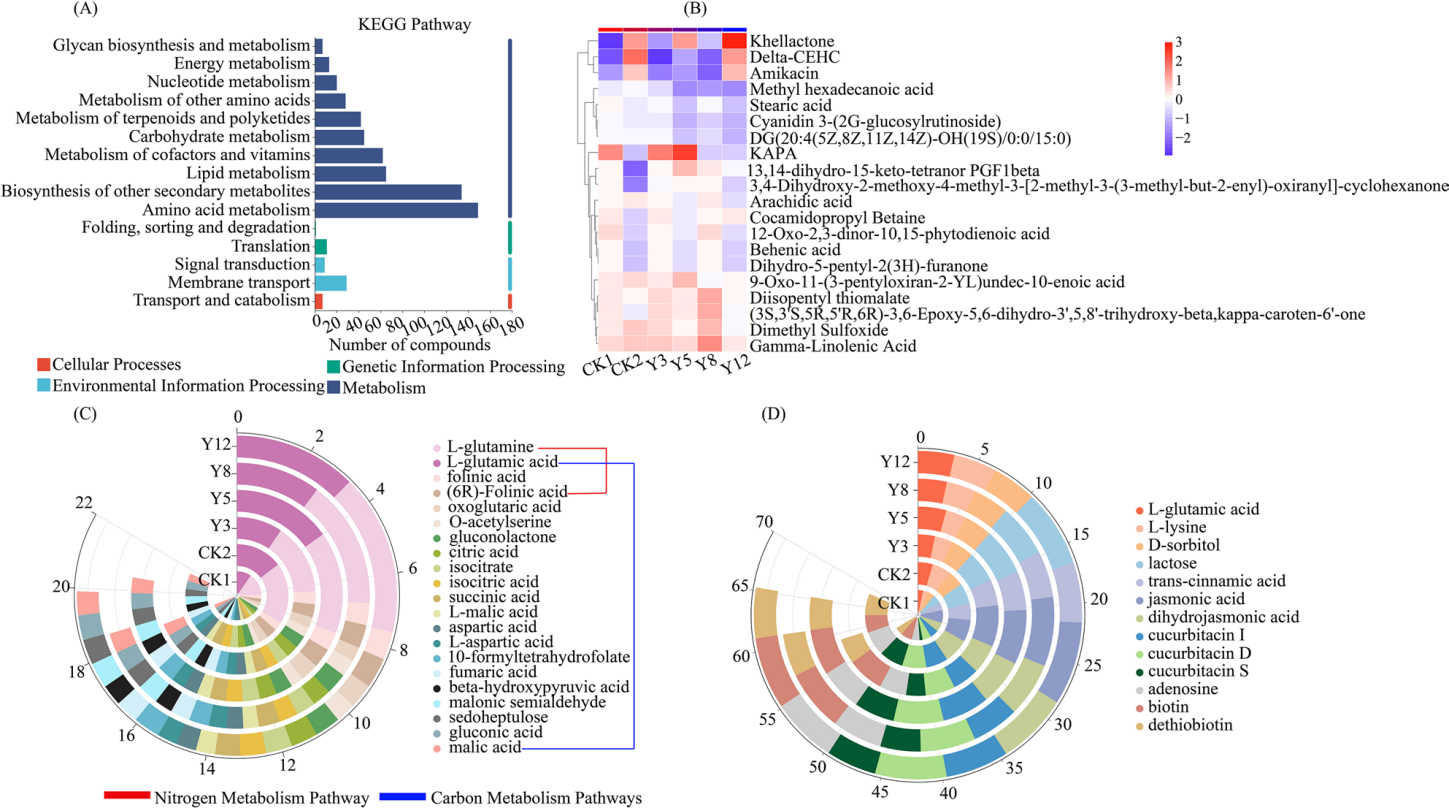
Analysis of soil metabolites in citrus orchards. **A** the relative abundances of level 2 KEGG pathways; **B** Relative Abundance of the Top 20 Metabolites; **C** the metabolites associated with carbon and nitrogen metabolism pathways; **D** The relative abundance of metabolites related to the transitional trophic modes of fungi

KEGG pathway annotation categorized the functional potential into four Level 1 groups: metabolism, environmental information processing, genetic information processing, and cellular processes (Fig. 3A). At Level 2, amino acid metabolism had the highest number of compounds, followed by biosynthesis of other secondary metabolites; in contrast, glycan biosynthesis and metabolism and energy metabolism had the lowest representation (Fig. 3A). Analysis of the top 20 metabolites revealed a clear connection between carbon cycling metabolites and terrace age: metabolites associated with the carbon cycle, including methyl hexadecanoic acid, stearic acid, arachidic acid, behenic acid, gamma-linolenic acid, and cocamidopropyl betaine, showed abundances consistent with cellulose degradation, a key carbon function. Specifically, these metabolites were the lowest in Y5 and highest in Y12 (Fig. 3B).

Within the nitrogen metabolism pathway, four metabolites were annotated, primarily L-glutamic acid and L-glutamine. L-glutamic acid peaked at Y5, while L-glutamine and folinic acid peaked at the oldest terrace age, Y12. Correspondingly, (6R)-Folinic acid showed its maximum abundance in the non-terraced control, CK2. The carbon metabolism pathway featured more compounds, with fermentation intermediates like oxoglutaric acid, L-malic acid, aspartic acid, fumaric acid, and beta-hydroxypyruvic acid all peaking in Y5. Conversely, O-acetylserine and gluconolactone had the lowest abundance in Y5 but peaked in Y8 (Fig. 3C). The abundances of metabolites associated with transitional fungal trophic modes also varied across the chronosequence (Fig. 3D). L-glutamic acid and cucurbitacins (I, D, and S) had the highest abundance in Y5, suggesting an abundant energy supply for saprophytic or pathogenic fungi. Metabolites, such as L-lysine, jasmonic acid, biotin, and dethiobiotin reached their lowest relative abundance at Y5. Notably, biotin and dethiobiotin peaked at Y8; D-sorbitol and jasmonic acid peaked early in Y3; and L-lysine, trans-cinnamic acid, dihydrojasmonic acid, and adenosine had the highest abundances in Y12. Furthermore, L-lysine, trans-cinnamic acid, dihydrojasmonic acid, and adenosine had the highest abundances in Y12. Moreover, lactose abundance progressively increased with terrace age (Fig. 3D).

### Correlations between soil properties and microbial communities

**Fig. 4:**
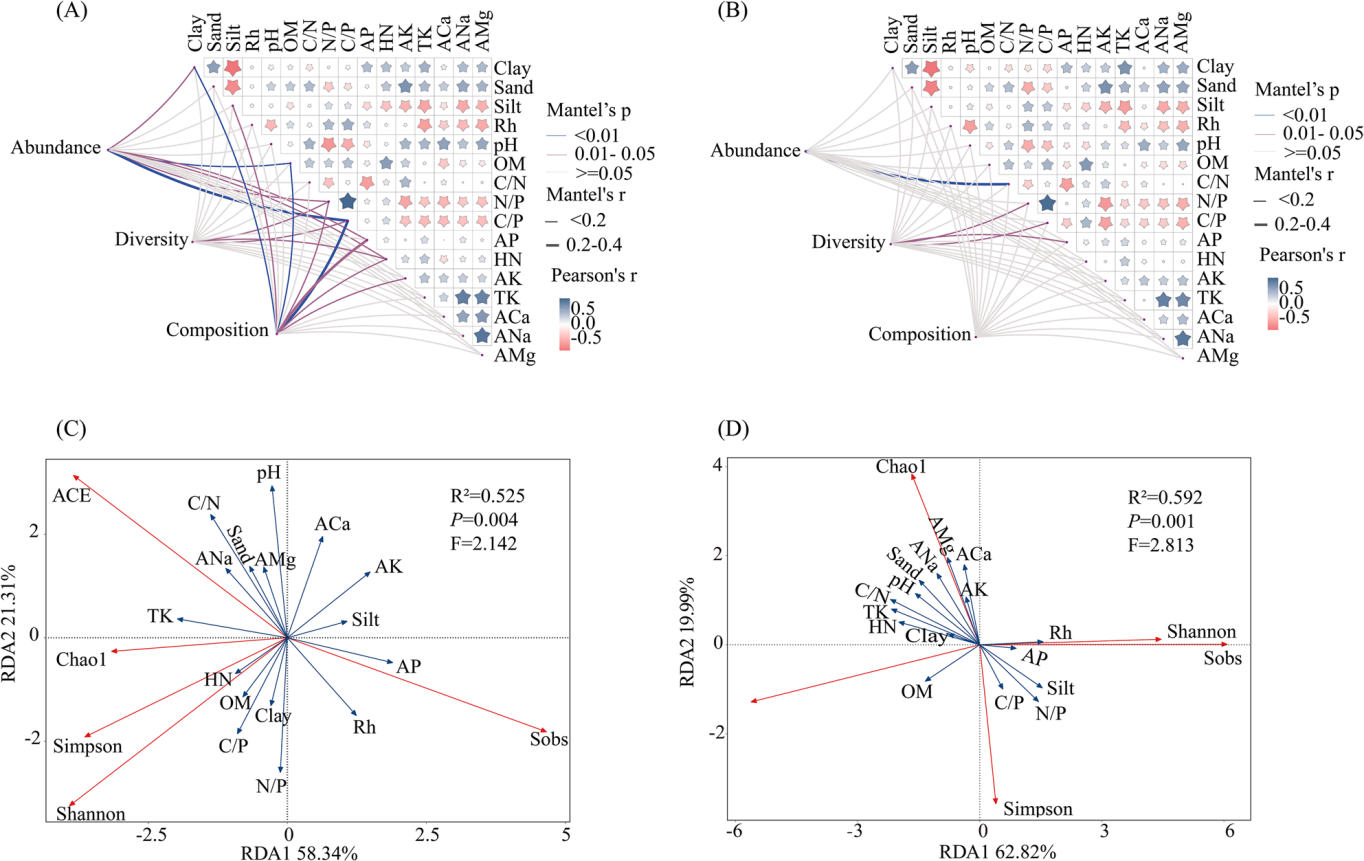
Correlation analysis of soil microbial community composition, abundance, diversity and soil properties in citrus orchards. **A**, **C** Bacteria, **B**,** D** Fungi

The interplay between soil properties and microbial communities was systematically evaluated through Mantel tests and RDA. For bacterial communities, community abundance exhibited significant positive correlations with AP, clay content, OM, and C/P ratio Community diversity (Shannon index) was influenced by N/P, HN, and silt content, while community composition (Sobs index) showed strong associations with C/P, AP, and AK. Notably, pH and TK had no significant impact on bacterial parameters (Fig. 4A, Table S2). Fungal communities display pronounced stoichiometric ratio dependence: richness was linked to soil C/N, and diversity correlated with N/P, C/P, and AP. Other soil properties exerted minimal effects on fungal dynamics (Fig. 4B, Table S2).

RDA further quantified these relationships between soil properties and microbial community. The first two RDA axes collectively explained 79.65% of the total variance in bacterial communities (RDA1: 58.34%, RDA2: 21.31%), with a reliable model fit (R^2^ = 0.525, *p* = 0.004, F = 2.142). Specifically, bacterial Shannon, Simpson, and Chao 1 indices were closely correlated with stoichiometric ratios (N/P, C/P) and OM; ACE index showed strong links to soil C/N ratio, soil pH, and TK; Sobs index was mainly driven by AP and Rh. These results confirmed that soil stoichiometric ratios, nutrient availability, and soil texture were the key environmental factors regulating bacterial community diversity and abundance (Fig. 4C). Corresponding fungal RDA revealed even stronger explanatory power, as the first two axes accounted for 82.81% of fungal community variance (RDA1: 62.82%, RDA2: 19.99%) with a highly significant model fit (R^2^ = 0.592, *p* = 0.001, F = 2.813). Fungal community abundance (Chao1) was primarily regulated by TK, C/N, and sand; Shannon and Sobs indices were driven by Rh and AP, while Simpson index was associated with C/P and N/P ratios. This indicated that fungal communities responded to a broader set of soil factors (including TK and sand) than bacterial communities, with nutrient availability and soil texture jointly shaping their dynamics (Fig. 4D).

**Fig. 5:**
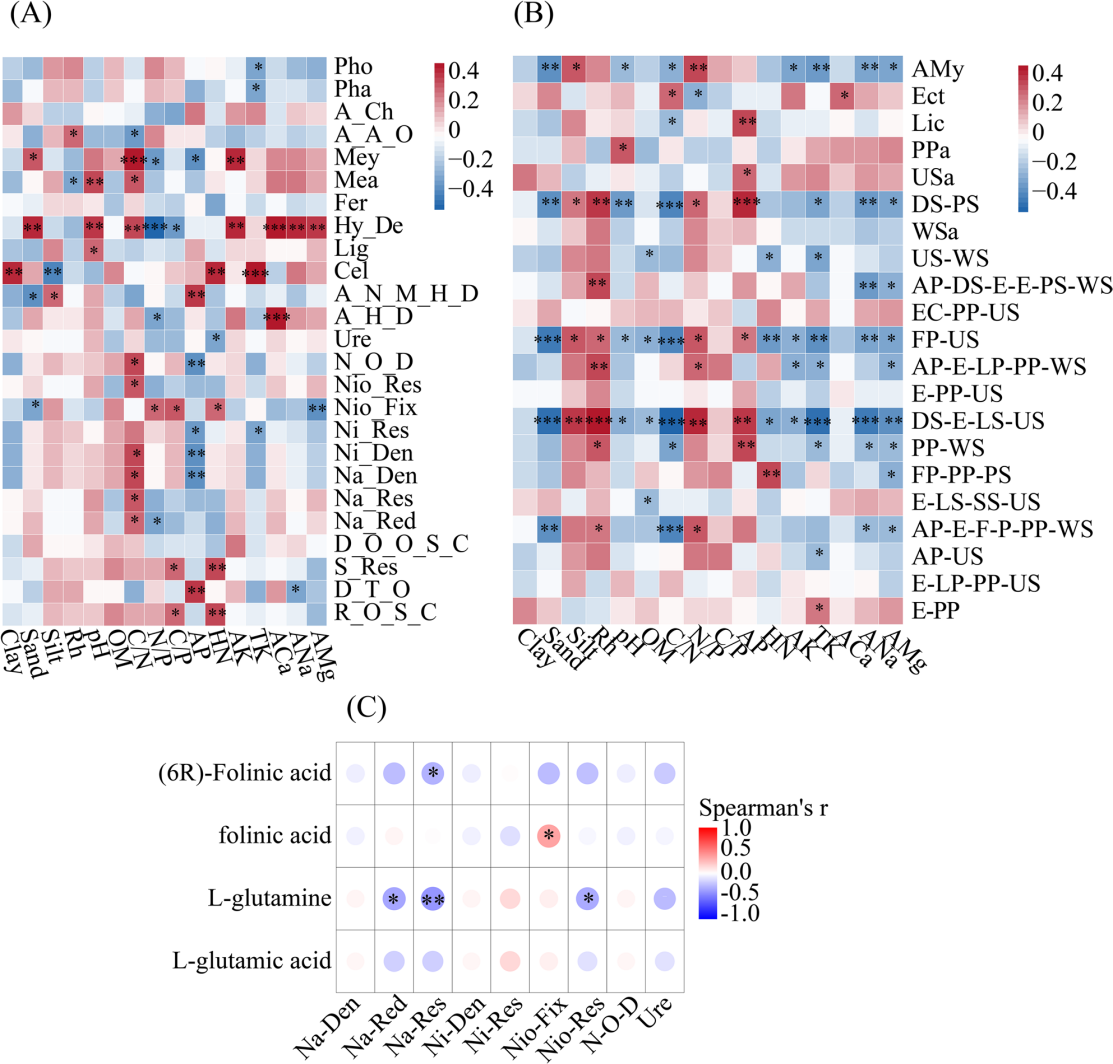
Correlation heat map of soil properties, microbial functions and nitrogen metabolites in citrus orchards. **A** bacteria, **B** fungi, **C** soil nitrogen metabolites and microbial functional prediction

Correlations analysis between soil physicochemical factors and the predicted relative abundance of bacterial community potential functional capabilities showed that soil TK was a key factor affecting the potential energy source of soil bacteria; soil C/N and AP were key factors associated with potential bacterial nitrogen cycling functions; pH and C/N were critical for bacterial carbon cycle functions; and sulfur cycle functional capabilities were mainly linked to C/P. With increasing terracing age, the potential abundance of sulfur-oxidizing bacteria decreased (Fig. 5A), which was consistent with the declining trend of soil C/P. The potential relative abundance of functional capabilities related to cellulolysis, hydrocarbon_degradation, aromatic_hydrocarbon_degradation, and aerobic_chemoheterotrophy increased with terrace age, while the potential relative abundance of methylotrophy-related functional capabilities decreased (*p* < 0.05). Potential nitrite_denitrification, nitrate_denitrification, nitrate_respiration, and nitrate_reduction were positively correlated with soil C/N but negatively correlated with soil AP, potential nitrogen_fixation, nitrate_respiration, nitrate_reduction, and nitrite_denitrification were positively correlated with soil organic matter and C/P, but negatively correlated with soil AP.

To determine the relationship between potential soil fungal functional guilds and environmental factors in citrus orchards, a heat map correlation analysis was carried out (Fig. 5B). The results showed that potential fungal parasite-undefined saprotroph and dung saprotroph-ectomycorrhizal-litter saprotroph-undefined saprotroph exhibited the highest number of significant correlations with soil physicochemical properties, followed by the potential dung saprotroph-plant saprotroph and arbuscular mycorrhizal. Significant correlations were observed between potential specific fungal functional guilds and soil properties: potential lichenised fungi were significantly positively correlated with soil AP (*p* = 0.008), but significantly negatively correlated with soil C/N (*p* = 0.042); potential undefined saprotroph was significantly positively correlated with soil AP (*p* = 0.027); potential endophyte-litter saprotroph-soil saprotroph-undefined saprotroph exhibited a significant negative correlation with soil OM (*p* = 0.042); potential animal pathogen-undefined saprotroph was significantly negatively correlated with soil TK (*p* = 0.036); potential endophyte-plant pathogen was significantly positively correlated with soil TK (*p* = 0.047); potential plant pathogen was significantly positively correlated with soil pH (*p* = 0.036); and potential fungal parasite-plant pathogen-plant saprotroph was significantly positively correlated with soil HN (*p* = 0.008) but negatively correlated with soil AMg (*p* = 0.027).

Correlation analysis between potential bacterial functional capabilities and metabolites showed that L-glutamine was significantly negatively correlated with nitrate_reduction, nitrate_respiration, and nitrogen_respiration (*p* < 0.05) (Fig. 5C). The potential relative abundance of nitrate_reduction, nitrate_respiration, and nitrogen_respiration were the highest in Y5, while L-glutamine peaked in Y12. Folate acid showed a significant positive correlation with potential nitrogen_fixation (*p* < 0.05). (6R)-Folate acid exhibited a significant negative correlation with potential nitrate_respiration (*p* < 0.05). Within carbon metabolism pathways, multiple metabolites, including (6R)-Folate acid, L-glutamic acid, isocitric acid, succinic acid, and L-aspartic acid, exhibited a significant negative correlation with potential hydrocarbon_degradation (*p* < 0.05), while showing a significant positive correlation with potential methanotrophy (*p* < 0.05) (Fig. S3A). Folate acid, citric acid, and sedoheptulose showed a significant positive correlation with potential cellulolysis (*p* < 0.05). In contrast, malic acid exhibited a significant negative correlation with potential cellulolysis (*p* < 0.05). Key fermentation intermediates—oxoglutaric acid, gluconolactone, and succinic acid—showed significant positive correlations with potential fermentation (*p* < 0.05) (Fig. S3A).

FUNGuild functional prediction revealed significant correlations between metabolites and potential fungal functional guilds (Fig. S3B). L-lysine exhibited a significant positive correlation with potential dung saprotroph-plant saprotroph, fungal parasite-undefined saprotroph, and plant pathogen-wood saprotroph (*p* < 0.05), while showing a significant negative correlation with potential endophyte-lichen parasite-plant pathogen-undefined saprotroph and ectomycorrhizal fungi (*p* < 0.05). Potential endophyte-plant pathogen-undefined saprotroph showed a significant negative correlation with cucurbitacin I and cucurbitacin D (*p* < 0.05), and a significant positive correlation with biotin (*p* < 0.05) (Fig. S3B).

### Co-occurrence network analysis and community assembly mechanisms of soil microbial communities

#### Co-occurrence network analysis

**Fig. 6:**
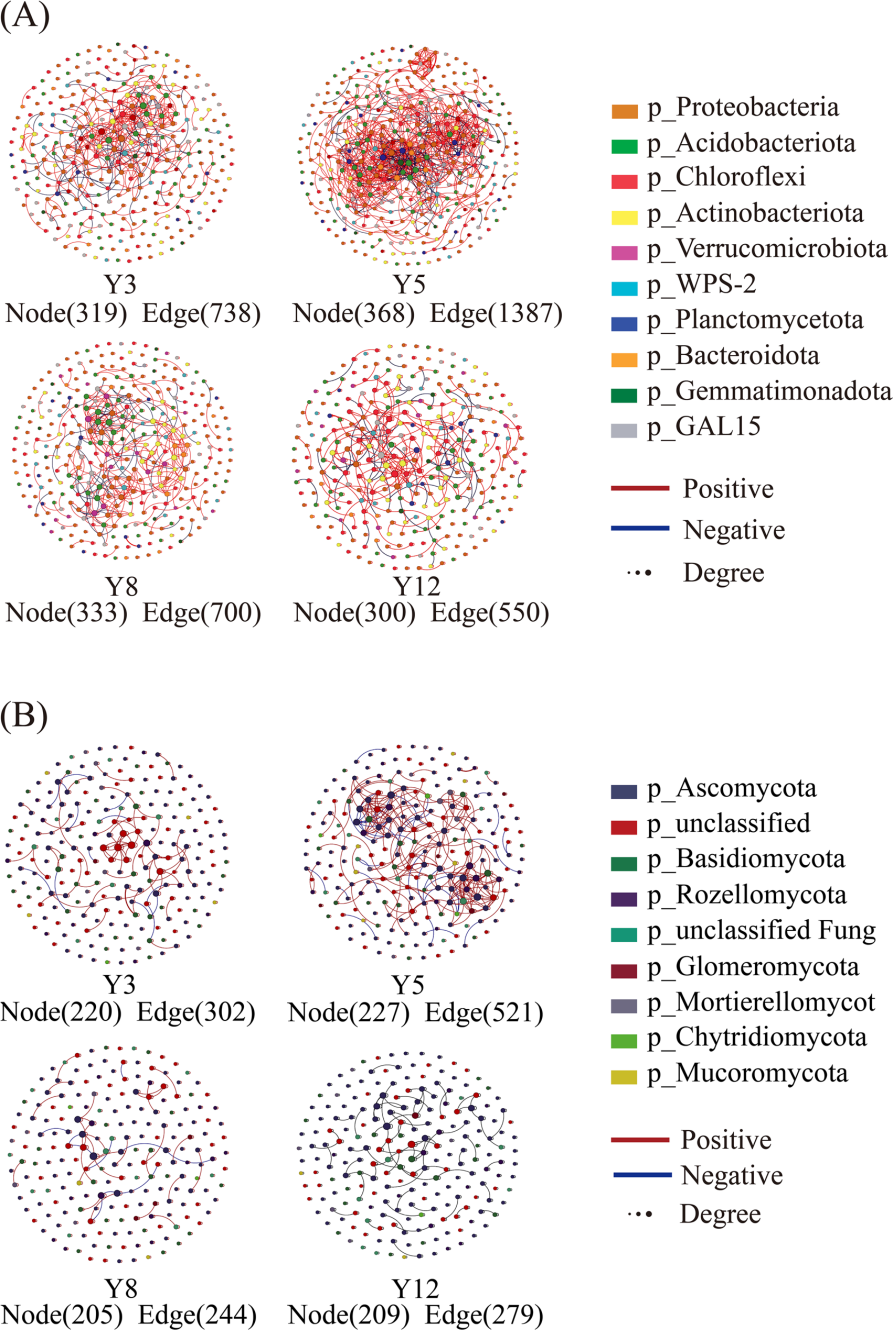
Soil microbial network structure in citrus orchards. Y3-Y12: terraces aged 3–12 years. **A** bacteria, **B** fungi. Different colors represent network modules, each node represents an individual OTU and the size of node represent the degree

To investigate microbial interaction dynamics under terracing, we analyzed co-occurrence networks through Spearman correlations (Fig. 6, Table S3). From Y3 to Y12, the number of Acidobacteriota nodes in microbial co-occurrence networks first increased then decreased, while their interactions with other microbial groups showed a trend of initial strengthening followed by weakening. Bacterial networks in Y5 demonstrated maximal structural complexity, with 368 nodes, 1,387 edges, and an average degree of 7.430—values significantly higher than those in younger or older terraced citrus orchards (Table S3). Modularity (0.626) and clustering coefficients (0.696) suggested moderate network integration, contrasting sharply with the fragmented topology observed in Y12 (modularity: 0.879; clustering: 0.779). Fungal networks mirrored this pattern, with Y5 exhibiting peak connectivity (521 edges, average degree 4.581) but reduced modular organization. Strikingly, negative interaction ratios reached 14.85% (bacteria) and 8.64% (fungi) in Y5, indicating intensified niche competition during intermediate terracing stages. Network complexity metrics (nodes, edges, clustering) followed a hump-shaped trajectory, peaking at Y5 before declining in older systems (Fig. 6).

Node roles were classified based on within-module (*Zi*) and among-module (*Pi*) connectivity. Bacterial communities comprised 18 modular hubs, 1 network hub, and 14 connectors, while fungal networks contained 8 modular hubs, 2 network hubs, and 76 connectors (Fig. S4). Over 96% of bacterial nodes (840/873) and 84% of fungal nodes (455/539) functioned as peripherals, whereas keystone taxa—including 18 bacterial modular hubs and 8 fungal modular hubs—highlighted taxa critical for maintaining network stability and function.

### Mechanisms of soil microbial community assembly in citrus orchards in the context of terracing

**Fig. 7:**
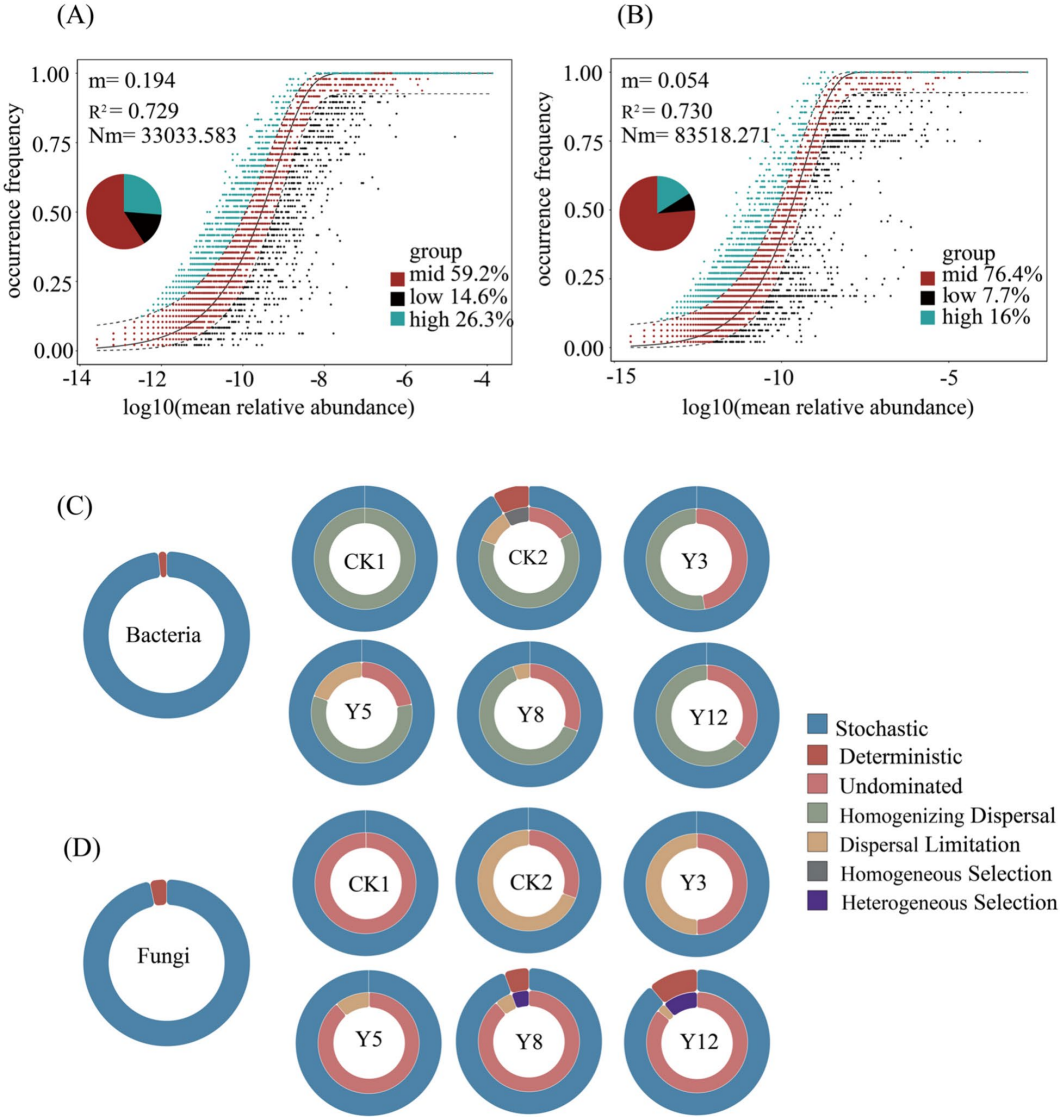
Assembly processes of soil microbial communities in citrus orchards. **A** Bacteria; **B** Fungi. CK1: natural forest; CK2: the non-terraced sloped farmland; Y3-Y12: terraces aged 3–12 years

The neutral model based on neutral theory was used to fit the frequency of occurrence of different OTUs to explore the mechanisms driving differences in the distributional characteristics of soil microbial communities in citrus orchards across terrace ages. The results showed that stochastic processes dominated microbial assembly, as evidenced by strong fits of the neutral community model (NCM; R^2^ = 72.9% for bacteria, 73% for fungi; Fig. 7A, B). Bacterial migration rates (m = 0.194) exceeded those of fungi (m = 0.054), reflecting that the assembly of fungal communities is subject to stronger dispersal limitation. Bacterial and fungal mobility was the highest in CK1 and lowest in CK2, and bacterial mobility was greater than fungal mobility in both land-use (Fig. S5). The results obtained based on the null model and Bray–Curtis indicated that the stochastic process can better explain the proportion of microbial community variations than the deterministic process, which supports the results of the neutral community model (Fig. 7C, D). Stochastic processes accounted for > 96% of community variation, with bacterial assembly driven by homogenizing dispersal and fungal dynamics by undominated processes within stochastic processes (e.g., drift, weak selection) (Fig. 7C, D). Deterministic selection emerged only in fungal communities beyond 8 years, likely due to heterogeneous soil nutrient gradients.

These findings highlight that mid-term terraced citrus orchards (Y5) optimize microbial connectivity, whereas prolonged terracing (> 8 years) favors deterministic filtering in fungi. The dominance of stochastic processes challenges assumptions of deterministic control in agricultural systems, emphasizing the need for age-specific management strategies in karst agriculture.

**Fig. 8:**
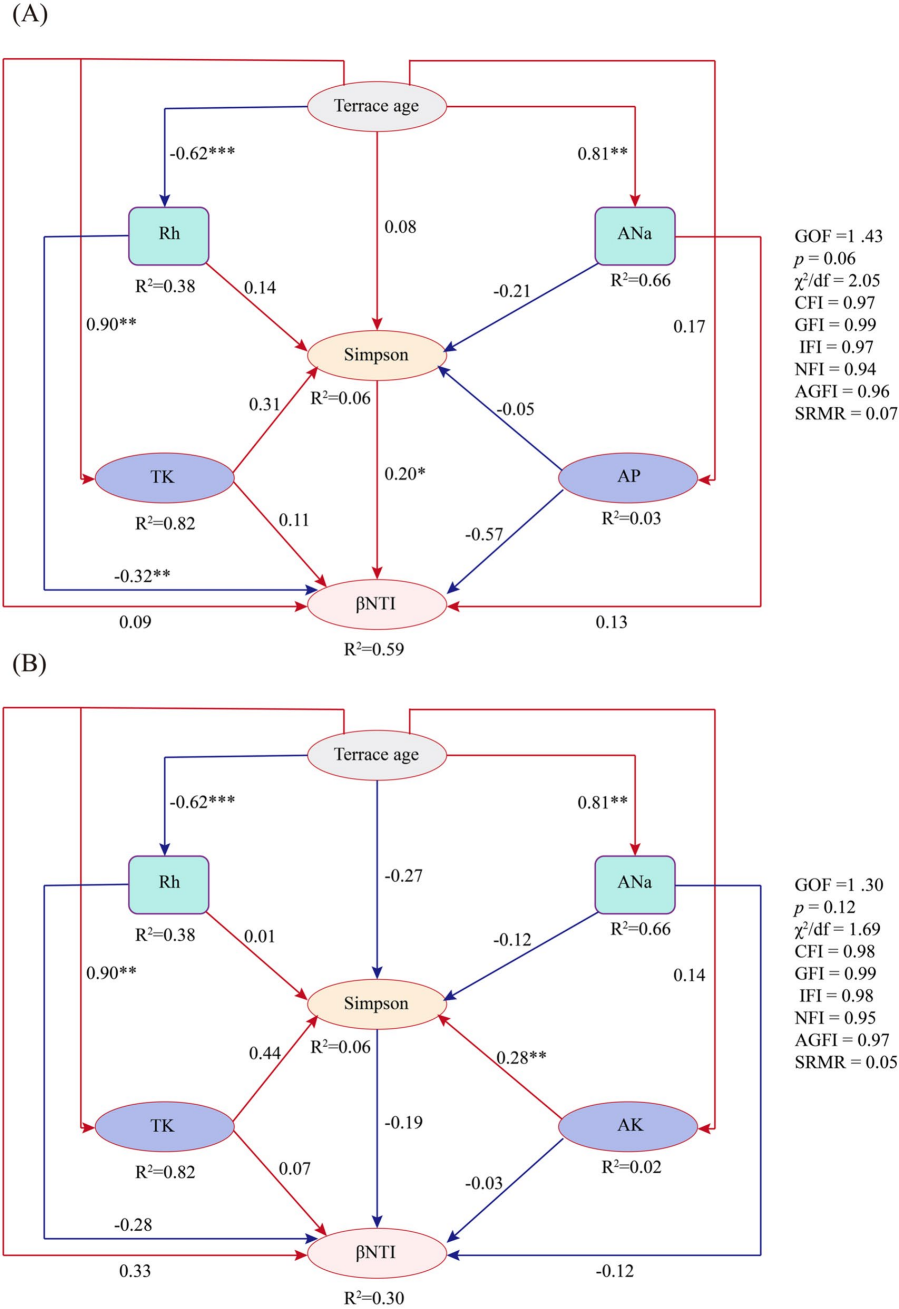
Piecewise structural equation model (piecewise SEM) shows the direct effects of environmental variables, microbial diversity, terrace age, and their indirect effects on phylogenetic turnover within different terraces ages. **A** Bacteria, **B** Fungi. Numbers above the arrows indicate path coefficients. R.^2^ values represent the proportion of variance explained by each variable. Solid red and solid blue arrows indicate positive and negative relationships. * *p* < 0.05, ** *p* < 0.01, *** *p* < 0.001

SEM was employed to assess causal relationships among multiple variables and evaluate competing hypotheses regarding microbial assembly pathways Specifically, SEM elucidated the direct and indirect effects of terrace age, soil properties (e.g., ANa, TK, AP), microbial diversity, and βNTI on community assembly. For bacterial communities, terrace age (*r* = 0.094), soil ANa (*r* = 0.125), TK (*r* = 0.106), and the Simpson index (*r* = 0.201, *p* < 0.05) exerted direct effects on phylogenetic turnover (βNTI). The terrace age further influenced bacterial assembly through the shifts of soil moisture (Rh: *r* = − 0.619, *p* < 0.001), ANa (*r* = 0.81, *p* < 0.001), AP (*r* = 0.171), and TK (*r* = 0.904, *p* < 0.001). Indirect pathways mediated through soil microorganisms were also observed for Rh (*r* = 0.143), ANa (*r* = − 0.205), AP (*r* = − 0.054), and TK (*r* = 0.313) (Fig. 8A).

In fungal communities, terrace age (*r* = 0.329) and soil TK (*r* = 0.071) directly drove phylogenetic turnover (βNTI). Indirect effects were detected for Rh (*r* = − 0.619, *p* < 0.001), ANa (*r* = 0.81, *p* < 0.001), AK (*r* = 0.145), and TK (*r* = 0.904, *p* < 0.001), with soil microorganisms acting as mediators (*r* = − 0.271). Notably, AK (*r* = 0.279, *p* < 0.05) indirectly affected fungal assembly via microbial interactions, whereas Rh (*r* = 0.006), ANa (*r* = − 0.119), and TK (*r* = 0.071) exerted negligible effects (Fig. 8B).

## Discussion

### Microbial community dynamics across terrace ages

To distinguish between low-yielding citrus plants and terrace effect, this study used non-terraced sloping farmland (CK2) as a control. Notably, terrace implementation did not alter the dominant microbial phyla but induced shifts in their relative abundances: CK2 exhibited the lowest relative abundance of Proteobacteria, while Y3 showed 1.65 times that of CK2, which decreased with terrace aging. Chloroflexi exhibited the highest relative abundance in CK2 and the lowest in Y3, though it increased with terrace aging (Fig. 2A). These shifts may be linked to the erosion-control benefits of terracing. By reducing soil loss, terracing helps create a more stable rhizosphere and improves conditions for citrus growth. Citrus plants may secrete numerous compounds via their roots to recruit diverse microorganisms for environmental adaptation. These substances not only promote microbial growth but also inhibit certain microorganisms, which may be one of the reasons for the changes in the relative abundance of microbial communities. It is particularly noteworthy that the increase in abundance of the Chloroflexi (includes oligotrophic microorganisms) in the later stages may suggest that as the terrace ecosystem develops, the soil environmental conditions slowly change to favor the enrichment of oligotrophic microorganisms. This shift likely explains the observed changes in microbial community abundance.

Soil microorganisms are of vital importance to the nutrient cycle, especially for carbon, nitrogen, and phosphorus. They promote plant growth by decomposing organic matter and enhancing the plant’s disease resistance. Their interaction with plant roots enhances the plant’s ability to acquire nutrients and its resilience to adverse environments. Specifically, soil bacterial communities in terraced citrus orchards exhibited distinct age-dependent compositional shifts, Proteobacteria, Acidobacteria, and Actinobacteria emerged as the dominant phyla, in line with previous studies of karst [43, 44], including in the microbial community in the rhizosphere of citrus [45]. Changes in the abundance of these dominant phyla may influence plant ammonia uptake and amino acid synthesis, affecting nutrient availability and stress resistance. As terraced citrus orchards age, the loss of soil organic matter leads to a decrease in the abundance of Proteobacteria, which weakens their oxidation function and limits the supply of available nitrogen and AP. This in turn restricts the absorption and utilization of these nutrients by citrus plants and their growth potential [46]. Actinomycetes may enhance citrus resistance to multiple plant pathogens by producing antimicrobial metabolites. Meanwhile, Proteobacteria and Actinobacteria can enhance plant absorption and transport of ammonia, promoting amino acid synthesis [47]. Plants recruit oligotrophs through metabolism to facilitate the absorption of nutrients, such as N, P and S. Acidobacteria, oligotrophic specialists crucial for nutrient cycling (degrading recalcitrant organic matter), maintained a stable abundance across chronosequences [48]. This may provide a sustained supply of available nutrients for citrus under various soil fertility conditions and support the continuous growth of plants [49]. Chloroflexi, which adapts to nutrient-limited environments, exhibited an increase in relative abundance from (value in Y3) to 10.64% in 12-year terraces (Y12), and has the ability to utilize complex organic compounds increased with the age of citrus cultivation, and did not only help stabilize terrace soils and reduce root exposure and erosion damage, but also enhanced organic matter decomposition and regulated the carbon pool and nutrient pool accessible to citrus roots [50]. The bacteria alpha diversity peaked in the intermediate ages (5–8 years). Higher microbial diversity helps establish a more balanced rhizosphere microbial community, enhancing soil nutrient cycling efficiency, promoting soil health, and suppressing plant pathogens, creating an optimal soil conditions for high citrus yield and quality [51]. These shifts were linked to soil environment: moderate nitrogen inputs increased bacterial abundance, whereas excessive nitrogen suppressed microbial growth, in line with previous findings that high nitrogen availability often impairs soil microbial function [52]. In addition, environmental heterogeneity, particularly sediment content variations by terrace implementation, modulated community structure by shaping soil physical properties (e.g., porosity, water-holding capacity) [51], indirectly influencing citrus root function.

The rich diversity of fungi may help maintain healthy ecological functions, including promoting nutrient cycling, organic matter decomposition, and plant productivity. In citrus rhizosphere soils, fungal communities were dominated by Ascomycota, Basidiomycota, and Rozellomycota, which together accounted for 75.95–86.75% of all fungal sequences (Fig. 2B). This phylum-level composition aligns with global soil fungal biogeography patterns and mirrors observations from other karst ecosystems [53], highlighting the ecological consistency of fungal community structure in degraded karst landscapes under terrace management. Ascomycota can degrade persistent organic matter such as lignin. As the age of terraced citrus orchards increases, Ascomycota accumulates due to the input of litters (e.g., citrus leaves and roots) [54]; this enrichment may enhance the efficiency of organic matter decomposition and alleviate nutrient limitation in older terraced citrus orchards, partly compensating for the deficiency in basic soil fertility and sustaining the long-term nutrition of citrus plants [55]. Ascomycetes exhibit stress resistance and produce secondary metabolites that inhibit other microorganisms. Additionally, they generate various antimicrobial agents that help protect plants from pathogens. Basidiomycota plays a vital role in supporting citrus plants to adapt to environmental stresses, playing a vital role in the decomposition of lignin-rich plant litter and converting these substrates to provide carbon, nitrogen, and other nutrients for plant growth. Its stable heat tolerance and drought resistance characteristics are key to coping with seasonal droughts in karst regions [47, 56]. Additionally, numerous Basidiomycota fungi can form symbiotic ectomycorrhizal relationships with citrus root systems, influencing root architecture and promoting deeper and denser root growth. This enhances water and N uptake, improving plant resilience under adverse conditions and boosting nutrient acquisition capacity [57]. Further, the ACE/Chao1 indices showed positive correlations with pH, C/N, TK, and AMg (*p* < 0.05), suggesting that nutrient redistribution under terrace management restructured the fungal community structure; this reorganization may affect the symbiotic (mycorrhizal) and saprophytic networks that are crucial for water absorption and organic nutrients acquisition by citrus plants and affect the plant’s ability to access resources and withstand stress.

Compared with citrus orchards planted on a flat terrain [58], those planted on our terraced system exhibited unique peaks in α-diversity during the Y5-Y8 period, a phenomenon that has not been reported in conventional orchards. This implies that the distinct microtopography and soil–water conservation environment resulting from terracing may support a divergent pattern of microbial community assembly compared to conventional management. This unique community dynamic may directly influence the soil nutrient retention capacity of the system. We hypothesize that this stage represents a transient optimal state of resource balance, which is crucial for maintaining soil nutrient retention and cycling functions in terraced citrus orchards. Therefore, the synergistic strategy combining strategic microtopography optimization with inoculation of erosion-resistant microbial communities is expected to serve as a key strategy for enhancing efficiency and stabilizing soil. Applying strategic microtopography optimization (e.g., adjusting terrace slopes, constructing micro-reservoirs, or utilizing plant windbreaks) can enhance water infiltration, intercept runoff pathways, and reduce soil erosion [59, 60]. This may also alleviate the nutrient decline observed in older terraced citrus orchards and support more resilient microbial communities (e.g., Chloroflexi). This strategy can be coupled with the inoculation of erosion-resistant microbial consortia (e.g., containing arbuscular mycorrhizal fungi and growth-promoting bacteria capable of exopolysaccharide secretion), which have been shown to enhance soil aggregation and stability [61, 62]. These approaches can jointly strengthen the biogeochemical cycling functions of terraced systems, providing a microbiome-based strategy to support both agricultural output and ecological resilience in karst landscapes.

However, it must be acknowledged that this study has several limitations must be considered. First, we did not directly measure microbial functional activity, and microbial functions prediction is limited by the scope of research methods. All discussions regarding microbial functions in this paper are based on the taxonomic composition derived from 16S rRNA gene and ITS sequencing data and on functional predictions using databases (e.g., FAPROTAX/FUNGuild) rather than direct measurements (e.g., metagenomics or metatranscriptomics). Accordingly, the predicted functional traits of these microorganisms represent potential rather than actually measured microbial activities. Although we attempted to cross-validate this predictive framework using soil metabolomics data, the functional potential inferred from phylogeny may still deviate from the actual in situ activity of microbial communities. Therefore, the inferred functional dynamics should be regarded as a reasonable hypothesis based on niche theory, which needs to be verified through direct methods such as metagenomic, metatranscriptomics, or targeted enzyme activity assays. A second limitation arises from the strong correlation between terrace age and citrus tree age in our sampling design. Because terraces and orchards were established at the same time, the observed changes in microbial communities likely result from a combination of soil habitat modification due to terracing and the progressive development of the citrus rhizosphere. This collinearity makes it difficult to isolate the individual effects of terrace engineering from those associated with root system maturation. Therefore, we still observed strong associations of microbial diversity, abundance, and community structure with soil nutrient stoichiometry (C:N:P). Significant differences were noted between terraced citrus orchards and non-terraced slope orchards, suggesting that the overall changes in soil physicochemical properties were driven by terrace management rather than isolated rhizosphere effects from a single plant species.

### Drivers of soil microbial community dynamics

Soil microbial communities exhibit high sensitivity to environmental shifts, driven by multifaceted interactions among soil properties, climatic variables, vegetation characteristics, and rhizosphere processes [63]. In terraced citrus orchards, terracing duration significantly altered soil physicochemical parameters (*p* < 0.05), creating distinct microhabitats for microbial assembly. RDA identified pH, TK, and C/N as pivotal drivers of microbial community structure (Fig. 4C, D). Specifically, bacterial assemblages responded strongly to AP, pH, and stoichiometric ratios (N/P, C/P, C/N), whereas fungal communities were primarily governed by HN, TK, and C/N. These findings align with previous reports emphasizing pH and nutrient stoichiometry as key determinants of bacterial diversity [64]. Notably, calcium-mediated pH regulation in karst systems plays a synergistic role in enhancing microbial diversity by buffering soil acidity and promoting nutrient mobilization. Bacterial abundance and diversity exhibited a unimodal pattern across terrace ages, closely mirroring trends observed in soil silt content. This phenomenon may arise from enhanced microhabitat heterogeneity in coarser-textured soils [65], where reduced resource competition promotes microbial colonization. Conversely, fungal communities displayed unique sensitivity to sand fraction, a trait potentially linked to the porous architecture and moisture retention characteristics of karst soils. AP and ACa emerged as critical regulators of bacterial composition, while fungal abundance positively correlated with HN, AMg, and ANa [66]. Karst-specific geochemical features further shaped these patterns: magnesium accumulation with terrace age (mediated by texture, precipitation, and fertilization) and dual limiting effects of C/P on microbial metabolism collectively constrained ecological functions in sinkhole microhabitats [67]. The view that the C/P stoichiometry regulates the metabolic allocation of microorganisms has been confirmed; that is, phosphorus plays a dominant role in constructing nitrogen cycling communities [68], and the abundance of sulfur-oxidizing bacteria declined with terrace age, aligning with the declining trend of soil C/P ratio. This change indicates that terrace construction causes microbial metabolism to shift toward the nitrogen cycle. Moreover, nitrogen cycling processes (e.g., denitrification and nitrogen fixation) are closely related to C/N, AP, and soil organic matter. Higher nitrogen availability seems to reduce nitrogen loss driven by denitrification, which may be because microorganisms preferentially assimilate nitrogen for growth. This pattern has also been found in karst ecosystems where nitrogen enrichment inhibits nitrification recovery [69]. Conversely, organic matter promoted nitrogen transformation by supplying carbon, alleviating nitrogen limitation, and modulating phosphorus supply, consistent with its identified role as a key driver of N mineralization in karst soils [69]. The high-calcium environment of karst soils acts as a strong filtering mechanism, favoring calcium-tolerant microorganisms while excluding sensitive species and reducing nutrient bioavailability. Low phosphorus conditions intensify phosphorus competition, promoting the enrichment of specific phosphorus-solubilizing microorganisms but suppressing overall community biomass and diversity. Highly heterogeneous terraced landscapes also fragment microhabitats, fostering locally specialized communities and weakening microbial connectivity at the landscape level. This highlights that microbial assembly patterns from general agricultural soils cannot be directly applied to karst ecosystems, underscoring the value of targeted research for karst agricultural soil management.

### Functional shifts and assembly mechanisms of soil microbial communities

The dynamic interplay between microbial community composition and functional diversity represents a critical focus in soil ecology. Alterations in microbial community structure directly modulate ecosystem functions, particularly nutrient cycling [70], and these processes are closely linked to plant growth, nutrient acquisition, and yield stability [71]. Our study based on high-throughput sequencing of bacterial 16S rRNA and fungal ITS genes combined with functional prediction, revealed that the age of terraced citrus orchards regulates microbial potential functional capabilities, influencing the nutritional status and stress resistance of citrus plants. The potential carbon- and nitrogen-cycling capabilities (e.g., nitrate reducers and nitrogen respirers) exhibited an initial increase followed by decline with terrace aging, whereas potential sulfur-cycling capabilities (e.g., sulfur oxidizers) continuously decreased (Fig. S2). This divergence likely reflects shifts in soil nutrient availability during terrace development. Nitrogen, a key element affecting plant growth and quality, is typically recycled by nitrogen-metabolizing microbes in the rhizosphere, enhancing the uptake of plant-available forms. The potential nitrogen cycling capacity (e.g., nitrogen fixation) peaked at Y5, can regulate nutrient utilization in citrus plants, ensuring efficient soil nitrogen transformation during this stage, and provide a foundation for the efficient utilization of nitrogen fertilizer by citrus root systems and promotion of yield. The related metabolites are of great significance in regulating the interaction between plants and microorganisms. The metabolite L-glutamine exhibits a negative correlation with potential nitrogen cycling capacity, suggesting that metabolite composition may constrain microbial function by inhibiting the accumulation of certain related functional capacities [39]. In addition, acidic substances (e.g., folinic acid) may impact microbial community diversity and composition indirectly by altering soil microenvironments (e.g., soil pH), potentially enhancing potential bacterial nitrogen_fixation functional capabilities to improve citrus nitrogen supply under nitrogen-limited conditions. With changes in nutrient levels during the aging process of terraces, the potential functional capabilities of oligotrophic sulfur-oxidizing bacteria decline [7]. Although sulfur is a secondary nutrient element for citrus, the reduction of cycling functional capabilities can weaken citrus stress resistance [72, 73]. The strong negative interactions are often regarded as indicators of heightened resource competition, leading to reduced network stability under environmental disturbances [74, 75]. Microbial network analysis revealed that Y5 exhibited the highest network complexity and a high proportion of negative interactions (compared to other years). This indicates that age can affect microbial resilience against external disturbances. In this study, microbial network complexity and potential nitrogen cycling function capacities peaked at Y5 (Fig. S2A), suggesting that this stage may represent a unique ecological equilibrium. This is likely due to the fact that the balanced soil resource conditions (e.g., optimal C/N and C/P ratios) support both high microbial diversity and functional specialization [76, 77]. The synergy between complex network structure and enhanced predicted nutrient cycling potential likely promotes more efficient carbon and nitrogen transformations in the soil, maximizing nutrient supply to support the growth and development of citrus plants [78, 79]. In Y12, P limitation drives microbial metabolic strategies to shift from nutrient acquisition to survival maintenance, resulting in a decline in the predicted functional capacities such as sulfide respiration. The potential multifunctional trophic strategies of fungal communities are a key feature for their adaptation to the heterogeneous soil environment of terrace orchards, and their potential trophic flexibility can effectively buffer abiotic stresses, such as drought and nutrient fluctuations, maintaining a stable rhizosphere environment for citrus growth [80, 81]. Root exudate metabolites mediate plant-soil feedback mechanisms by selectively shaping the microbial community (recruiting beneficial groups or suppressing harmful ones). Similar effects have been reported in studies on both monoculture of grapevines and the rhizosphere of medicinal plants [82, 83]. Intriguingly, potential ectomycorrhizal fungal abundance inversely correlated with soil total nitrogen [84, 85], suggesting that nitrogen enrichment may suppress certain symbiotic functions, altering the mycorrhizal benefits for P uptake and drought tolerance in citrus plants.

In the assembly patterns of microbial communities, deterministic and stochastic ecological processes are complementary and continuous. Community assembly analysis revealed that stochastic processes (e.g., stochastic births, deaths, and migration) dominated both bacterial (98.4%) and fungal (96.7%) communities, as evidenced by high neutral model fit (R^2^ > 0.70) and βNTI values, consistent with research on soil microorganisms in ryegrass systems, and also aligns with the results of relevant studies focusing on orchard ecosystems where stochastic processes are proven to be the dominant driver of soil microbial community assembly [85, 86]. In contrast, deterministic processes were found to dominate in rice and wheat systems [87]. Analysis based on the neutral community model showed that the bacterial communities exhibited higher migration rate (migration rate: 0.194 vs. 0.054 for fungi), suggesting a stronger potential influence of dispersal limitation on fungal community assembly relative to that of bacteria in this karst slope environment. This may be partly due to the smaller cell size of bacteria, which enhances passive diffusion; fungi grow more slowly and face more intense competition, thereby enhancing deterministic filtering [22, 88–90]. Of course, the community construction process is diverse, and the construction of the bacterial community may also be dominated by strong deterministic processes in other systems [91, 92]. Additionally, terrace implementation may reduce the deterministic environmental filtering of bacteria, allowing stochastic colonization to play a more vital role. Concurrently, fungal assembly showed greater sensitivity to deterministic filtering (e.g., soil TK, AK), indicating that deterministic environmental selection exerts measurable effects alongside stochasticity. Such deterministic signals may relate to functional specialization within fungal niches, such as complex organic matter decomposition and symbiotic relationships [22, 93]. Soil TK content increased with terraced citrus orchard age, providing a greater reserve source of AK. Although stochastic processes (e.g., dispersal limitation) exerted decisive dominance over fungal community assembly across all terraced citrus orchards, the relative contribution of deterministic processes—particularly heterogeneous selection—exhibited an increasing trend in older citrus orchards (Y8–Y12), becoming more pronounced than that in younger citrus orchards, where AK explained 27.9% of the βNTI variation (Fig. 8B). This indicates that the aging process progressively selects K-adapted specialists, consistent with the increased abundance of *Saitozyma* (K-transporter gene carrier). In contrast to typical agricultural systems, where deterministic processes dominate microbial assembly [94], stochastic process prevail in karst terrace soils, albeit through distinct mechanisms in bacterial and fungal communities. Homogenizing dispersal contributes more to the assembly of bacterial communities, while the undominated process is more important in fungal communities; dispersal limitation is only significant in some fungal communities (CK2, Y3). This discrepancy may stem from the unique high heterogeneity of karst environments: a shallow soil depth leads to an insufficient matrix continuity, inhibiting large-scale directed microbial diffusion; fragmented microhabitats partition microbial communities into isolated “microhabitat islands,” making migration more dependent on stochastic events; and a high Ca^2^⁺ content in karst environments masks pH effects on communities through calcium buffering. Furthermore, as a high-calcium environment, karst regions harbor abundant genes associated with microbial growth and reproduction, thereby amplifying the influence of random processes on microbial community assembly [95]. Collectively, these factors constrain microbial migration through both physical and chemical mechanisms, establishing critical conditions for stochastic processes to dominate community assembly.

SEM further identified terrace age, soil moisture, and potassium dynamics as pivotal abiotic regulators of assembly pathways. Notably, pH, a well-documented community driver [11], showed negligible influence here, possibly masked by calcium buffering in karst soil. Moreover, peak network complexity at Y5 coincided with the highest nitrogen-cycling potential, suggesting that co-occurrence patterns may support functional resilience. This link has not been reported before in terraced systems (Fig. S6).

## Conclusion

This study highlights that terrace age is a key factor shaping soil microbial communities in citrus orchards on karst sloping land. Across a 12-year chronosequence, microbial abundance did not undergo significant compositional shifts. Instead, microbial diversity was primarily correlated with soil stoichiometric ratios, while also showing a strong association with predicted microbial functions. Bacterial communities exhibited a potential prioritization of nitrogen cycling processes such as nitrification and denitrification, followed by carbon metabolism. In contract, fungal communities displayed potential transitional strategies consistent with their role in organic matter decomposition. Notably, microbial community assembly was overwhelmingly governed by stochastic processes, which accounted for more than 96% of the observed patterns (bacteria: 98.4%; Fungi: 96.5%). Homogenizing dispersal emerged as the dominant force shaping bacterial communities, whereas fungal community dynamics were influenced by undominated processes, including drift and weak selection. These findings emphasize the critical role of stochastic processes in shaping karst soil microbiomes, suggesting that effective microbial management strategies should prioritize dispersal dynamics and niche-neutrality interactions. In addition, the aggregation of potential microbial functional capabilities in Y5 and the increased complexity of microbial co-occurrence networks suggest a potential for more efficient carbon and nitrogen transformation in terrace rhizosphere soil. We recommend prioritizing the optimization of terrace rotation cycles during this critical 5-year transition window to promote microbial-driven nitrogen cycling. ‌Meanwhile,‌ terrace management should maintain a balance between agricultural productivity and ecological functions, thereby facilitating more sustainable nutrient management practices in fragile karst agroecosystems. For future research, integrating metagenomic and metatranscriptomic approaches will enable direct assessment of microbial functional activity. Conducting field-controlled experiments could further clarify the independent contributions of terrace engineering, citrus root systems, and management practices to microbial community structure, ultimately revealing more precise driving mechanisms underlying microbiome assembly in these agroecosystems.

## Supplementary Information

Supplementary Material 1.

## Data Availability

The microbial datasets provided in this study are available in an online repository. The name and login number of the repository can be found below: https://www.ncbi.nlm.nih.gov/, [PRJNA1247777](https:/www.ncbi.nlm.nih.gov/bioproject/PRJNA1247777) and [PRJNA1247886](https:/www.ncbi.nlm.nih.gov/bioproject/PRJNA1247886).

## References

[1] Wu Q, Wang L. Suitability of agronomic water saving in karst areas and its enlightenment in the karst desertification control. Heliyon. 2024;10(11):e32568. 10.1016/j.heliyon.2024.e32568. 38933953 10.1016/j.heliyon.2024.e32568 PMC11201120

[2] Dai Y, Song Y, Zhang J, Zhao B, Li L, Huang Z, et al. Phylogenetic relationships versus environmental impacts on the distribution and traits of Laureae (Lauraceae) species within and outside karst tiankengs. Am J Bot. 2025;112(5):e70032. 10.1002/ajb2.70032. 40255204 10.1002/ajb2.70032

[3] Cao L, Wang S, Peng T, Cheng Q, Zhang L, Zhang Z, et al. Monitoring of suspended sediment load and transport in an agroforestry watershed on a karst plateau, Southwest China. Agric Ecosyst Environ. 2020;299:106976. 10.1016/j.agee.2020.106976.

[4] Dai YX, Huang JP, Zhang JJ, Yang H. Phylogenetic relationships of *Smilax astrosperma* (Smilacaceae) with other *Smilax* species and its potential distribution patterns in karst areas of south China. Nord J Bot. 2025;2025(6):e04551. 10.1111/njb.04551.

[5] Deng C, Zhang G, Liu Y, Nie X, Li Z, Liu J, et al. Advantages and disadvantages of terracing: a comprehensive review. Int Soil Water Conserv Res. 2021;9(3):344–59. 10.1016/j.iswcr.2021.03.002.

[6] Colozzi Filho AB, Bertagnoli A, Menoncin J, Oliveira I, Campana G, Machineski G, Barbosa. Terracing Reduces Arbuscular Mycorrhizal fungi Spore Loss through Surface Runoff. Brazilian Archives of Biology and Technology. 2024;67;e24230801. 10.1590/1678-4324-PSSM-2024230801

[7] Qiu Y, Fu Q, Yang Y, Zhao J, Li J, Yi F, et al. Soil and stone terraces offset the negative impacts of sloping cultivation on soil microbial diversity and functioning by protecting soil carbon. J Environ Manage. 2024;369:122339. 10.1016/j.jenvman.2024.122339. 39222589 10.1016/j.jenvman.2024.122339

[8] Ning D, Wang Y, Fan Y, Wang J, Van Nostrand JD, Wu L, et al. Environmental stress mediates groundwater microbial community assembly. Nat Microbiol. 2024;9(2):490–501. 10.1038/s41564-023-01573-x. 38212658 10.1038/s41564-023-01573-x

[9] Stegen JC, Lin X, Fredrickson JK, Konopka AE. Estimating and mapping ecological processes influencing microbial community assembly. Front Microbiol. 2015;6:370. 10.3389/fmicb.2015.00370. 25983725 10.3389/fmicb.2015.00370 PMC4416444

[10] Vellend M. Conceptual synthesis in community ecology. Q Rev Biol. 2010;85(2):183–206. 10.1086/652373. 20565040 10.1086/652373

[11] Tripathi BM, Stegen JC, Kim M, Dong K, Adams JM, Lee YK. Soil pH mediates the balance between stochastic and deterministic assembly of bacteria. ISME J. 2018;12(4):1072–83. 10.1038/s41396-018-0082-4. 29515169 10.1038/s41396-018-0082-4 PMC5864241

[12] Zhang L, Zuo Q, Cai H, Li S, Shen Z, Song T. Fungicides reduce soil microbial diversity, network stability and complexity in wheat fields with different disease resistance. Appl Soil Ecol. 2024;201:105513. 10.1016/j.apsoil.2024.105513.

[13] Jiao S, Yang YF, Xu YQ, Zhang J, Lu YH. Balance between community assembly processes mediates species coexistence in agricultural soil microbiomes across eastern China. ISME J. 2020;14(1):202–16. 10.1038/s41396-019-0522-9. 31611655 10.1038/s41396-019-0522-9 PMC6908645

[14] Powell JR, Karunaratne S, Campbell CD, Yao HY, Robinson L, Singh BK. Deterministic processes vary during community assembly for ecologically dissimilar taxa. Nat Commun. 2015;6:8444. 10.1038/ncomms9444. 26436640 10.1038/ncomms9444 PMC4600744

[15] Fierer N, Leff JW, Adams BJ, Nielsen UN, Bates ST, Lauber CL, et al. Cross-biome metagenomic analyses of soil microbial communities and their functional attributes. Proc Natl Acad Sci U S A. 2012;109(52):21390–5. 10.1073/pnas.1215210110. 23236140 10.1073/pnas.1215210110 PMC3535587

[16] Allan E, Manning P, Alt F, Binkenstein J, Blaser S, Bluethgen N, et al. Land use intensification alters ecosystem multifunctionality via loss of biodiversity and changes to functional composition. Ecol Lett. 2015;18(8):834–43. 10.1111/ele.12469. 26096863 10.1111/ele.12469 PMC4744976

[17] Liu W, Graham EB, Zhong L, Zhang J, Li S, Lin X, et al. Long-term stochasticity combines with short-term variability in assembly processes to underlie rice paddy sustainability. Front Microbiol. 2020;11:873. 10.3389/fmicb.2020.00873. 32499764 10.3389/fmicb.2020.00873 PMC7243440

[18] Peng Z, Wang Z, Liu Y, Yang T, Chen W, Wei G, et al. Soil phosphorus determines the distinct assembly strategies for abundant and rare bacterial communities during successional reforestation. Soil Ecol Lett. 2021;3(4):342–55. 10.1007/s42832-021-0109-z.

[19] Kang LY, Chen LY, Zhang DY, Peng YF, Song YT, Kou D, et al. Stochastic processes regulate belowground community assembly in alpine grasslands on the Tibetan Plateau. Environ Microbiol. 2022;24(1):179–94. 10.1111/1462-2920.15827. 34750948 10.1111/1462-2920.15827

[20] Jiao S, Chu H, Zhang B, Wei X, Chen W, Wei G. Linking soil fungi to bacterial community assembly in arid ecosystems. iMeta. 2022;1(1):e2. 10.1002/imt2.2. 10.1002/imt2.2 PMC10989902 38867731

[21] Chen WD, Ren KX, Isabwe A, Chen HH, Liu M, Yang J. Stochastic processes shape microeukaryotic community assembly in a subtropical river across wet and dry seasons. Microbiome. 2019;7(1):138. 10.1186/s40168-019-0749-8. 31640783 10.1186/s40168-019-0749-8 PMC6806580

[22] Zhu G, Luan L, Zhou S, Dini-Andreote F, Bahram M, Yang Y, et al. Body size mediates the functional potential of soil organisms by diversity and community assembly across soil aggregates. Microbiol Res. 2024;282:127669. 10.1016/j.micres.2024.127669. 38442455 10.1016/j.micres.2024.127669

[23] Sanches VL, de Souza Mesquita LM, Viganó J, Contieri LS, Pizani R, Chaves J, et al. Insights on the extraction and analysis of phenolic compounds from citrus fruits: green perspectives and current status. Crit Rev Analyt Chem. 2024;54(5):1173–1199. 10.1080/10408347.2022.2107871. 10.1080/10408347.2022.2107871 35993795

[24] Zhu D, Cheng X, Li W, Niu F, Wen J. Temporal and spatial variation characteristics of water quality in the Middle and Lower Reaches of the Lijiang River, China and their responses to environmental factors. Int J Environ Res Public Health. 2022;19(13):8089. 10.3390/ijerph19138089. 35805749 10.3390/ijerph19138089 PMC9266160

[25] Gerasimova MI. Chinese soil taxonomy: between the American and the international classification systems. Eurasian Soil Sci. 2010;43(8):945–9. 10.1134/S1064229310080120.

[26] Wang R, Wang M, Wang J, Lin Y. Habitats are more important than seasons in shaping soil bacterial communities on the Qinghai-Tibetan Plateau. Microorganisms. 2021;9(8):1595. 10.3390/microorganisms9081595. 34442674 10.3390/microorganisms9081595 PMC8400953

[27] Yuan Y-H, Liu L-X, Wang L, Dong G-Z, Liu Y-G. Effects of different seasons on bacterial community structure in rose rhizosphere soil. Appl Microbiol Biotechnol. 2023;107(1):405–17. 10.1007/s00253-022-12290-6. 36418546 10.1007/s00253-022-12290-6

[28] Ai L, Wei M, Ma J, Dai Y, Zhang J, Chen F, et al. Occurrence patterns and ecological implications of microplastic contamination in citrus orchard soils on Karst sloping terrains, South China. J Hazard Mater. 2025;496:139391. 10.1016/j.jhazmat.2025.139391. 40753805 10.1016/j.jhazmat.2025.139391

[29] Zhao W, Wen M, Zhao C, Zhang S, Dou R, Liang X, et al. Warm temperature increments strengthen the crosstalk between roots and soil in the rhizosphere of soybean seedlings. Plants. 2023;12(24):4135. 10.3390/plants12244135. 38140462 10.3390/plants12244135 PMC10747358

[30] Fan Z, Lu S, Liu S, Li Z, Hong J, Zhou J, et al. The effects of vegetation restoration strategies and seasons on soil enzyme activities in the Karst landscapes of Yunnan, southwest China. J Forestry Res. 2020;31(5):1949–57. 10.1007/s11676-019-00959-0.

[31] Novák V, Hlaváčiková H. Soil-Water Content and Its Measurement. In: Novák V, Hlaváčiková H, editors. Applied Soil Hydrology. Cham: Springer International Publishing; 2019. p. 49–61.

[32] Hong S, Gan P, Chen A. Environmental controls on soil pH in planted forest and its response to nitrogen deposition. Environ Res. 2019;172:159–65. 10.1016/j.envres.2019.02.020. 30782535 10.1016/j.envres.2019.02.020

[33] Yu X, Yang J, Liu L, Tian Y, Yu Z. Effects of *Spartina alterniflora* invasion on biogenic elements in a subtropical coastal mangrove wetland. Environ Sci Pollut Res Int. 2015;22(4):3107–15. 10.1007/s11356-014-3568-2. 25233914 10.1007/s11356-014-3568-2

[34] Xu Y, Pu L, Zhang R, Zhu M, Zhang M, Bu X, et al. Effects of agricultural reclamation on soil physicochemical properties in the Mid-Eastern Coastal Area of China. Land. 2021;10(2):142. 10.3390/land10020142.

[35] Cicchella D, Ambrosino M, Albanese S, Guarino A, Lima A, De Vivo B, et al. Major elements concentration in soils. A case study from Campania Region (Italy). J Geochem Explor. 2023;247:107179. 10.1016/j.gexplo.2023.107179.

[36] Olsen SR, Sommers LE. Phosphorus. In: Page ALE, editor. Methods of Soil Analysis, Part 2: Chemical and Microbiological Properties. 1982. p. 403–430. 10.2134/agronmonogr9.2.2ed.c24.

[37] Zhang J. Influences of Protective Forest Construction on Soil Nutrient Dynamics. In: Zhang J, editor. Forestry Measures for Ecologically Controlling Non-point Source Pollution in Taihu Lake Watershed: China. Singapore: Springer; 2016. p. 179–193. 10.1007/978-981-10-1850-3_12.

[38] Mozaffari H, Moosavi AA, Dematte JAM. Estimating particle-size distribution from limited soil texture data: introducing two new methods. Biosyst Eng. 2022;216:198–217. 10.1016/j.biosystemseng.2022.02.007.

[39] Wang Y, Zhang H, Zhang Y, Fei J, Xiangmin R, Peng J, et al. Crop rotation-driven changes in rhizosphere metabolite profiles regulate soil microbial diversity and functional capacity. Agric Ecosyst Environ. 2023;358:108716. 10.1016/j.agee.2023.108716.

[40] Ai L, Dai Y, Chen F, Zhang J,Ma J, Bai K, et al. Dynamic changes of soil metabolite profiles during Moso bamboo (Phyllostachys edulis (Carrière) J. Houz.) expansion into adjacent planted forests in a typical karst river basin, South China. Appl Soil Ecol. 2024;203:105671. 10.1016/j.apsoil.2024.105671

[41] Wu S, Wu K, Shi L, Sun X, Tan Q, Hu C. Recruitment of specific microbes through exudates affects cadmium activation and accumulation in *Brassica napus*. J Hazard Mater. 2023;442:130066. 10.1016/j.jhazmat.2022.130066. 36193614 10.1016/j.jhazmat.2022.130066

[42] Kang H, Yu W, Dutta S, Gao H. Soil microbial community composition and function are closely associated with soil organic matter chemistry along a latitudinal gradient. Geoderma. 2021;383:114744. 10.1016/j.geoderma.2020.114744.

[43] Tang Q, Li Q, Tong L, Wu R, Xu J. Rhizospheric soil organic carbon accumulated but its molecular groups redistributed via rhizospheric soil microorganisms along multi-root *Cerasus humilis* plantation chronosequence at the karst rocky desertification control area. Environ Sci Pollut Res Int. 2023;30(28):72993–3007. 10.1007/s11356-023-27588-9. 37184792 10.1007/s11356-023-27588-9

[44] Jiang C, Sun X-R, Feng J, Zhu S-F, Shui W. Metagenomic analysis reveals the different characteristics of microbial communities inside and outside the karst tiankeng. BMC Microbiol. 2022;22(1):115. 10.1186/s12866-022-02513-1. 35473500 10.1186/s12866-022-02513-1 PMC9040234

[45] Castellano-Hinojosa A, Albrecht U, Strauss SL. Interactions between rootstocks and compost influence the active rhizosphere bacterial communities in citrus. Microbiome. 2023;11(1):79. 10.1186/s40168-023-01524-y. 37076924 10.1186/s40168-023-01524-y PMC10116748

[46] Castellano-Hinojosa A, Kanissery R, Strauss SL. Cover crops in citrus orchards impact soil nutrient cycling and the soil microbiome after three years but effects are site-specific. Biol Fertil Soils. 2023;59(6):659–78. 10.1007/s00374-023-01729-1.

[47] Jibola-Shittu MY, Heng Z, Keyhani NO, Dang Y, Chen R, Liu S, et al. Understanding and exploring the diversity of soil microorganisms in tea (*Camellia sinensis*) gardens: toward sustainable tea production. Front Microbiol. 2024;15:1379879. 10.3389/fmicb.2024.1379879. 38680916 10.3389/fmicb.2024.1379879 PMC11046421

[48] Sun H, Hu W, Dai Y, Ai L, Wu M, Hu J, et al. Moso bamboo (Phyllostachys edulis (Carrière) J. Houzeau) invasion affects soil microbial communities in adjacent planted forests in the Lijiang River basin, China. Front Microbiol. 2023;14:1111498. 10.3389/fmicb.2023.1111498. 10.3389/fmicb.2023.1111498 PMC9990415 36896433

[49] Gonçalves OS, Fernandes AS, Tupy SM, Ferreira TG, Almeida LN, Creevey CJ, et al. Insights into plant interactions and the biogeochemical role of the globally widespread Acidobacteriota phylum. Soil Biol Biochem. 2024;192:109369. 10.1016/j.soilbio.2024.109369.

[50] Huo J, Song B, Lin X, Riaz M, Zhao X, Liu S, et al. Ecological characteristics of sugar beet plant and rhizosphere soil in response to high boron stress: a study of the remediation potential. J Environ Manage. 2024;356:120655. 10.1016/j.jenvman.2024.120655. 38513589 10.1016/j.jenvman.2024.120655

[51] Wu H, Xu G, Yang R, Dai J, Al-Dhabi NA, Wang G, et al. Responses of soil antibiotic resistance genes to the decrease in grain size of sediment discharged into Dongting Lake, China. Sci Total Environ. 2024;953:176091. 10.1016/j.scitotenv.2024.176091. 39244058 10.1016/j.scitotenv.2024.176091

[52] Tang J, Su L, Fang Y, Wang C, Meng L, Wang J, et al. Moderate nitrogen reduction increases nitrogen use efficiency and positively affects microbial communities in agricultural soils. Agriculture. 2023;13(4):796. 10.3390/agriculture13040796.

[53] He B, Li Q, Li W, Zou S, Bai X, Chen Y. Dynamic changes of rhizosphere soil microbial community along a Karst coniferous plantation chronosequence. J Soil Sci Plant Nutr. 2024;24(4):7398–417. 10.1007/s42729-024-02048-6.

[54] Bhatti AA, Haq S, Bhat RA. Actinomycetes benefaction role in soil and plant health. Microb Pathog. 2017;111:458–67. 10.1016/j.micpath.2017.09.036. 28923606 10.1016/j.micpath.2017.09.036

[55] Shanmugam SG, Kingery WL. Changes in soil microbial community structure in relation to plant succession and soil properties during 4000 years of pedogenesis. Eur J Soil Biol. 2018;88:80–8. 10.1016/j.ejsobi.2018.07.003.

[56] Bui A, Orr D, Lepori-Bui M, Konicek K, Young H, Moeller HV. Soil fungal community composition and functional similarity shift across distinct climatic conditions. FEMS Microbiol Ecol. 2020;96(12):fiaa193. 10.1093/femsec/fiaa193. 10.1093/femsec/fiaa193 32960210

[57] Kuyper TW, Suz LM. Do ectomycorrhizal trees select ectomycorrhizal fungi that enhance phosphorus uptake under nitrogen enrichment? Forests. 2023;14(3):467. 10.3390/f14030467.

[58] Jin Y-b, Fang Z, Zhou X-b. Variation of soil bacterial communities in a chronosequence of citrus orchard. Ann Microbiol. 2022;72(1):21. 10.1186/s13213-022-01681-9.

[59] Deng K, Zhu Y, Liu Z, Sun G, Han X, Zheng H. Effects of crop growth and surface microtopography on runoff and soil losses in the red soil region of southern China. CATENA. 2024;238:107894. 10.1016/j.catena.2024.107894.

[60] Wei W, Pan D, Yang Y. Effects of terracing measures on water retention of *pinus tabulaeformis* forest in the dryland loess hilly region of China. Agric For Meteorol. 2021;308:108544. 10.1016/j.agrformet.2021.108544.

[61] Liu X, Mei S, Salles JF. Inoculated microbial consortia perform better than single strains in living soil: a meta-analysis. Appl Soil Ecol. 2023;190:105011. 10.1016/j.apsoil.2023.105011.

[62] Yusuf A, Jiang Y, Abdullahi A, Li M, Duan S, Zhang Y. Bacterial-fungal interactions in soil ecosystems: from biocontrol and niche partitioning to biogeochemical impacts. Fungal Ecol. 2025;78:101471. 10.1016/j.funeco.2025.101471.

[63] Tian C, Wu X, Bahethan B, Yang X, Yang Q, Wang X. Soil bacterial community characteristics and influencing factors in different types of farmland shelterbelts in the Alaer reclamation area. Front Plant Sci. 2024;15:1488089. 10.3389/fpls.2024.1488089. 39534107 10.3389/fpls.2024.1488089 PMC11555564

[64] Xiang J, Gu J, Wang G, Bol R, Yao L, Fang Y, et al. Soil pH controls the structure and diversity of bacterial communities along elevational gradients on Huangshan, China. Eur J Soil Biol. 2024;120:103586. 10.1016/j.ejsobi.2023.103586.

[65] Yang Y, Chen Q, Yu W, Shi Z. Estimating soil bacterial abundance and diversity in the Southeast Qinghai-Tibet Plateau. Geoderma. 2022;416. 10.1016/j.geoderma.2022.115807.

[66] Nopnakorn P, Zhang Y, Yang L, Peng F. Antarctic Ardley Island terrace — an ideal place to study the marine to terrestrial succession of microbial communities. Front Microbiol. 2023;14:942428. 10.3389/fmicb.2023.942428. 36814563 10.3389/fmicb.2023.942428 PMC9940900

[67] Jiang H, Chen X, Li Y, Chen J, Wei L, Zhang Y. Seasonal dynamics of soil microbiome in response to dry–wet alternation along the Jinsha River Dry-hot Valley. BMC Microbiol. 2024;24(1):496. 10.1186/s12866-024-03662-1. 39587503 10.1186/s12866-024-03662-1 PMC11587743

[68] Xiao D, Xiao L, Che R, Tan Y, Liu X, Yang R, et al. Phosphorus but not nitrogen addition significantly changes diazotroph diversity and community composition in typical karst grassland soil. Agric Ecosyst Environ. 2020;301:106987. 10.1016/j.agee.2020.106987.

[69] Hu P, Zhao Y, Xiao D, Xu Z, Zhang W, Xiao J, et al. Dynamics of soil nitrogen availability following vegetation restoration along a climatic gradient of a subtropical karst region in China. J Soils Sediments. 2021;21(6):2167–78. 10.1007/s11368-021-02915-0.

[70] Shao S, Li Y, Li Z, Ma X, Zhu Y, Luo Y, et al. Impact of tea tree cultivation on soil microbiota, soil organic matter, and nitrogen cycling in mountainous plantations. Agron. 2024;14(3):638. 10.3390/agronomy14030638.

[71] Gougoulias C, Clark JM, Shaw LJ. The role of soil microbes in the global carbon cycle: tracking the below-ground microbial processing of plant-derived carbon for manipulating carbon dynamics in agricultural systems. J Sci Food Agric. 2014;94(12):2362–71. 10.1002/jsfa.6577. 24425529 10.1002/jsfa.6577 PMC4283042

[72] Shekhawat K, Veluchamy A, Fatima A, García-Ramírez GX, Reichheld J-P, Artyukh O, et al. Microbe-induced coordination of plant iron–sulfur metabolism enhances high-light-stress tolerance of Arabidopsis. Plant Commun. 2024;5(11):101012. 10.1016/j.xplc.2024.101012. 38956873 10.1016/j.xplc.2024.101012 PMC11589330

[73] Asai T, Matsukawa T, Kajiyama Si. Metabolomic analysis of primary metabolites in citrus leaf during defense responses. J Biosci Bioeng. 2017;123(3):376–81. 10.1016/j.jbiosc.2016.09.013. 27789172 10.1016/j.jbiosc.2016.09.013

[74] Yuan MM, Guo X, Wu L, Zhang Y, Xiao N, Ning D, et al. Climate warming enhances microbial network complexity and stability. Nat Clim Chang. 2021;11(4):343–8. 10.1038/s41558-021-00989-9.

[75] Hernandez DJ, David AS, Menges ES, Searcy CA, Afkhami ME. Environmental stress destabilizes microbial networks. ISME J. 2021;15(6):1722–34. 10.1038/s41396-020-00882-x. 33452480 10.1038/s41396-020-00882-x PMC8163744

[76] Mooshammer M, Wanek W, Zechmeister-Boltenstern S, Richter AA. Stoichiometric imbalances between terrestrial decomposer communities and their resources: mechanisms and implications of microbial adaptations to their resources. Front Microbiol. 2014;5:22. 10.3389/fmicb.2014.00022. 24550895 10.3389/fmicb.2014.00022 PMC3910245

[77] Zechmeister-Boltenstern S, Keiblinger KM, Mooshammer M, Peñuelas J, Richter A, Sardans J, et al. The application of ecological stoichiometry to plant-microbial-soil organic matter transformations. Ecol Monogr. 2015;85(2):133–55. 10.1890/14-0777.1.

[78] Gardner JG, Schreier HJ. Unifying themes and distinct features of carbon and nitrogen assimilation by polysaccharide-degrading bacteria: a summary of four model systems. Appl Microbiol Biotechnol. 2021;105(21):8109–27. 10.1007/s00253-021-11614-2. 34611726 10.1007/s00253-021-11614-2

[79] Freilich S, Zarecki R, Eilam O, Segal ES, Henry CS, Kupiec M, et al. Competitive and cooperative metabolic interactions in bacterial communities. Nat Commun. 2011;2(1):589. 10.1038/ncomms1597. 22158444 10.1038/ncomms1597

[80] Cheng H, Giri B, Wu Q, Ying Z, Kuca K. Arbuscular mycorrhizal fungi mitigate drought stress in citrus by modulating root microenvironment. Arch Agro Soil Sci. 2021;68. 10.1080/03650340.2021.1878497.

[81] Cao JL, He WX, Zou YN, Wu QS. An endophytic fungus, *Piriformospora indica*, enhances drought tolerance of trifoliate orange by modulating the antioxidant defense system and composition of fatty acids. Tree Physiol. 2023;43(3):452–66. 10.1093/treephys/tpac126. 36263985 10.1093/treephys/tpac126

[82] Du H, Wang B, Dawood M, Qu P, Li W, Zhang L, et al. Root diameter-associated exudates drive the changes in rhizosphere microbial communities. J Soil Sci Plant Nutr. 2025;25(2):2438–50. 10.1007/s42729-025-02276-4.

[83] Song R, Lv B, He Z, Li H, Wang H. Rhizosphere metabolite dynamics in continuous cropping of vineyards: impact on microflora diversity and co-occurrence networks. Microbiol Res. 2025;296:128134. 10.1016/j.micres.2025.128134. 40068342 10.1016/j.micres.2025.128134

[84] Argiroff WA, Zak DR, Pellitier PT, Upchurch RA, Belke JP. Decay by ectomycorrhizal fungi couples soil organic matter to nitrogen availability. Ecol Lett. 2022;25(2):391–404. 10.1111/ele.13923. 34787356 10.1111/ele.13923

[85] Liu HQ, Li SC, Li HJ, Peng ZC. Soil pH determining the assembly processes of abundant and rare bacterial communities in response to cultivation modes in lemon farmlands. Plants. 2025;14(12):1852. 10.3390/plants14121852. 40573840 10.3390/plants14121852 PMC12196865

[86] Larsen S, Albanese D, Stegen J, Franceschi P, Coller E, Zanzotti R, et al. Distinct and temporally stable assembly mechanisms shape bacterial and fungal communities in vineyard soils. Microb Ecol. 2023;86(1):337–49. 10.1007/s00248-022-02065-x. 35835965 10.1007/s00248-022-02065-x PMC10293400

[87] Cui S, Xu S, Cao G, Zhu X. The long-term straw return resulted in significant differences in soil microbial community composition and community assembly processes between wheat and rice. Front Microbiol. 2025;16–2025. 10.3389/fmicb.2025.1533839. 10.3389/fmicb.2025.1533839 PMC11903465 40083788

[88] Lu M, Wang X, Li H, Jiao JJJ, Luo X, Luo M, et al. Microbial community assembly and co-occurrence relationship in sediments of the river-dominated estuary and the adjacent shelf in the wet season. Environ Pollut. 2022;308:119572. 10.1016/j.envpol.2022.119572. 35661808 10.1016/j.envpol.2022.119572

[89] Luan L, Jiang YJ, Cheng MH, Dini-Andreote F, Sui YY, Xu QS, et al. Organism body size structures the soil microbial and nematode community assembly at a continental and global scale. Nat Commun. 2020;11(1):6406. 10.1038/s41467-020-20271-4. 33335105 10.1038/s41467-020-20271-4 PMC7747634

[90] Wang WT, Sun ZH, Mishra S, Xia SW, Lin LX, Yang XD. Body size determines multitrophic soil microbiota community assembly associated with soil and plant attributes in a tropical seasonal rainforest. Mol Ecol. 2023;32(23):6294–303. 10.1111/mec.16585. 35770463 10.1111/mec.16585

[91] Chen QL, Hu HW, Yan ZZ, Li CY, Nguyen BAT, Sun AQ, et al. Deterministic selection dominates microbial community assembly in termite mounds. Soil Biol Biochem. 2021;152:108073. 10.1016/j.soilbio.2020.108073.

[92] Cao Y, Chai YF, Jiao S, Li XY, Wang XB, Zhang YN, et al. Bacterial and fungal community assembly in relation to soil nutrients and plant growth across different ecoregions of shrubland in Shaanxi, northwestern China. Appl Soil Ecol. 2022;173:104385. 10.1016/j.apsoil.2022.104385.

[93] Zinger L, Taberlet P, Schimann H, Bonin A, Boyer F, De Barba M, et al. Body size determines soil community assembly in a tropical forest. Mol Ecol. 2019;28(3):528–43. 10.1111/mec.14919. 30375061 10.1111/mec.14919

[94] Dai W, Liu Y, Yao D, Wang N, Shao J, Ye X, et al. Biogeographic distribution, assembly processes and potential nutrient cycling functions of myxobacteria communities in typical agricultural soils in China. Sci Total Environ. 2024;906:167255. 10.1016/j.scitotenv.2023.167255. 37741390 10.1016/j.scitotenv.2023.167255

[95] Long X, Li J, Liao X, Zhang W, Wang K, Zhao J. Linking microbial metabolism and ecological strategies to soil carbon cycle function in agroecosystems. Soil Tillage Res. 2025;251:106562. 10.1016/j.still.2025.106562.

