# Supplementary material for: Terracing influences soil microbial assembly in citrus orchards: stochastic processes dominate community dynamics in a karst sloping land
Source: BMC Microbiol. 2026 Feb 25;26:295. doi: 10.1186/s12866-026-04811-4 (PMC13041272; doi:10.1186/s12866-026-04811-4)
Supplement: Supplementary file 1 — Supplementary Material 1. [file 12866_2026_4811_MOESM1_ESM.pdf]

Terracing Influences Soil Microbial Assembly in Citrus Orchards: Stochastic Processes Dominate Community Dynamics in a Karst Sloping Land

Jiaojiao Zhang [1, 2 †], Yuxin Dai [1, 2 †], Adnan Mustafa [3], Liwen Li [2], Yuxuan Li [2], Tongfang Sun [1, 2], Minglei Chen [1, 2], Jiangming Ma [1, 2, *], Hao Yang [1, 2, *]

[1] Key Laboratory of Ecology of Rare and Endangered Species and Environmental Protection (Guangxi Normal University), Ministry of Education/Guangxi Key Laboratory of Landscape Resources Conservation and Sustainable Utilization in Lijiang River Basin, Guilin 541006, China.

[2] College of Life Science, Guangxi Normal University, Guilin 541006, China.

[3] Guangdong Provincial Key Laboratory of Applied Botany, South China Botanical Garden, Chinese Academy of Sciences, Guangzhou 510650, China

E-mail: yanghao_henry@gxnu.edu.cn

[†] Jiaojiao Zhang and Yuxin Dai contributed equally to this work.

* These authors are co-corresponding authors.

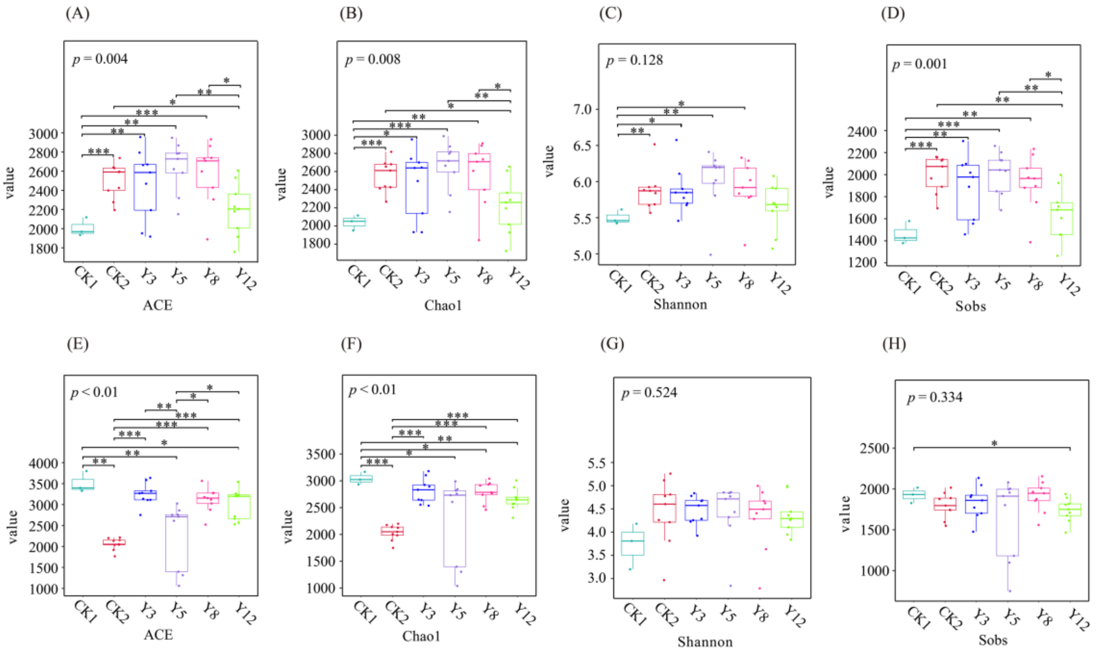

Fig. S1. α-Diversity dynamics of soil microbial communities. (**A**, **B**, **C**, **D**) Bacterial indices: ACE, Chao1, Shannon, and Sobs. (**E**, **F**, **G**, **H**). Fungal indices. * $p < 0.05$, ** $p < 0.01$, *** $p < 0.001$. CK1: Natural forest; CK2: the non-terraced sloped farmland; Y3-Y12: terraces aged 3-12 years.

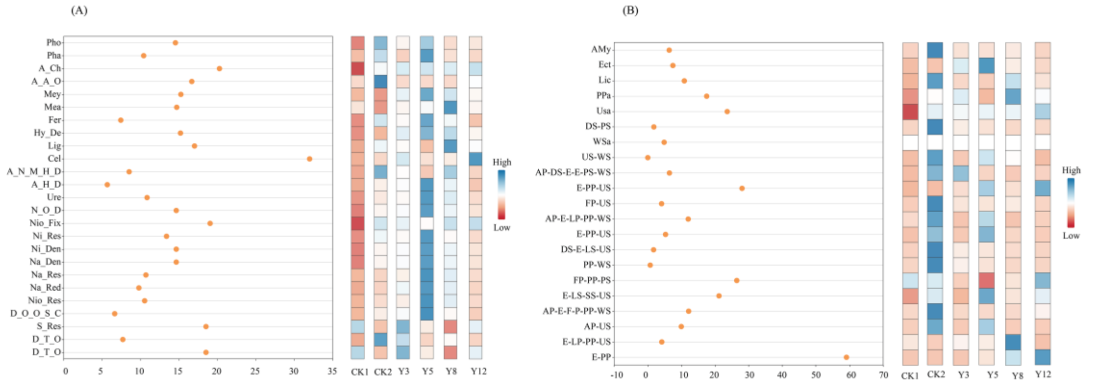

Fig. S2. Prediction of soil microbial functions in citrus orchards. (**A**) Bacteria, (**B**) Fungi. CK1: natural forest; CK2: the non-terraced sloped farmland; Y3-Y12: terraces aged 3-12 years. **A_A_O:** aerobic_ammonia_oxidation; **D_T_O:** dark_thiosulfate_oxidation; **R_O_S_C:** respiration_of_sulfur_compounds; **S_Res:** sulfate_respiration; **Mea:** methanotrophy; **Lig:** ligninolysis; **A_N_M_H_D:** aliphatic_non_methane_hydrocarbon_degradation; **D_O_O_S_C:** dark_oxidation_of_sulfur_compounds; **A_H_D:** aromatic_hydrocarbon_degradation; **Mey:** methylotrophy; **Hy_De:** hydrocarbon_degradation; **Pha:** photoautotrophy; **Na_Den:** nitrate_denitrification; **Ni_Den:** nitrite_denitrification; **N_O_D:** nitrous_oxide_denitrification; **Pho:** photoheterotrophy; **Ni_Res:** nitrite_respiration; **Fer:** fermentation; **Na_Res:** nitrate_respiration; **Nio_Res:** nitrogen_respiration; **Ure:** ureolysis; **Na_Red:** nitrate_reduction; **Nio_Fix:** nitrogen_fixation; **Cel:** cellulolysis; **A_Ch:** aerobic_chemoheterotrophy; **E-PP:** endophyte-plant pathogen; **US-WS:** undefined saprotroph-wood saprotroph; **Lic:** lichenized; **AP-E-LP-PP-WS:** animal pathogen-endophyte-lichen parasite-plant pathogen-wood saprotroph; **AP-US:** animal

pathogen-undefined saprotroph; **AP-E-F-P-PP-WS:** animal pathogen-endophyte-fungal parasite-plant pathogen-wood saprotroph; **DS-PS:** dung saprotroph-plant saprotroph; **EC-PP-US:** endomycorrhizal-plant pathogen-undefined saprotroph; **DS-E-LS-US:** dung saprotroph-ectomycorrhizal-litter saprotroph-undefined saprotroph; **FP-US:** fungal parasite-undefined saprotroph; **FP-PP-PS:** fungal parasite-plant pathogen-plant saprotroph; **Ect:** ectomycorrhizal; **WSa:** wood saprotroph; **PP-WS:** plant pathogen-wood saprotroph; **E-LP-PP-US:** endophyte-lichen parasite-plant pathogen-undefined saprotroph; **PPa:** plant pathogen; **AP-DS-E-E-PS-WS:** animal pathogen-dung saprotroph-endophyte-epiphyte-plant saprotroph-wood saprotroph; **E-LS-SS-US:** endophyte-litter saprotroph-soil saprotroph-undefined saprotroph; **E-PP-US:** endophyte-plant pathogen-undefined saprotroph; **AMy:** arbuscular mycorrhizal; Usa: undefined saprotroph

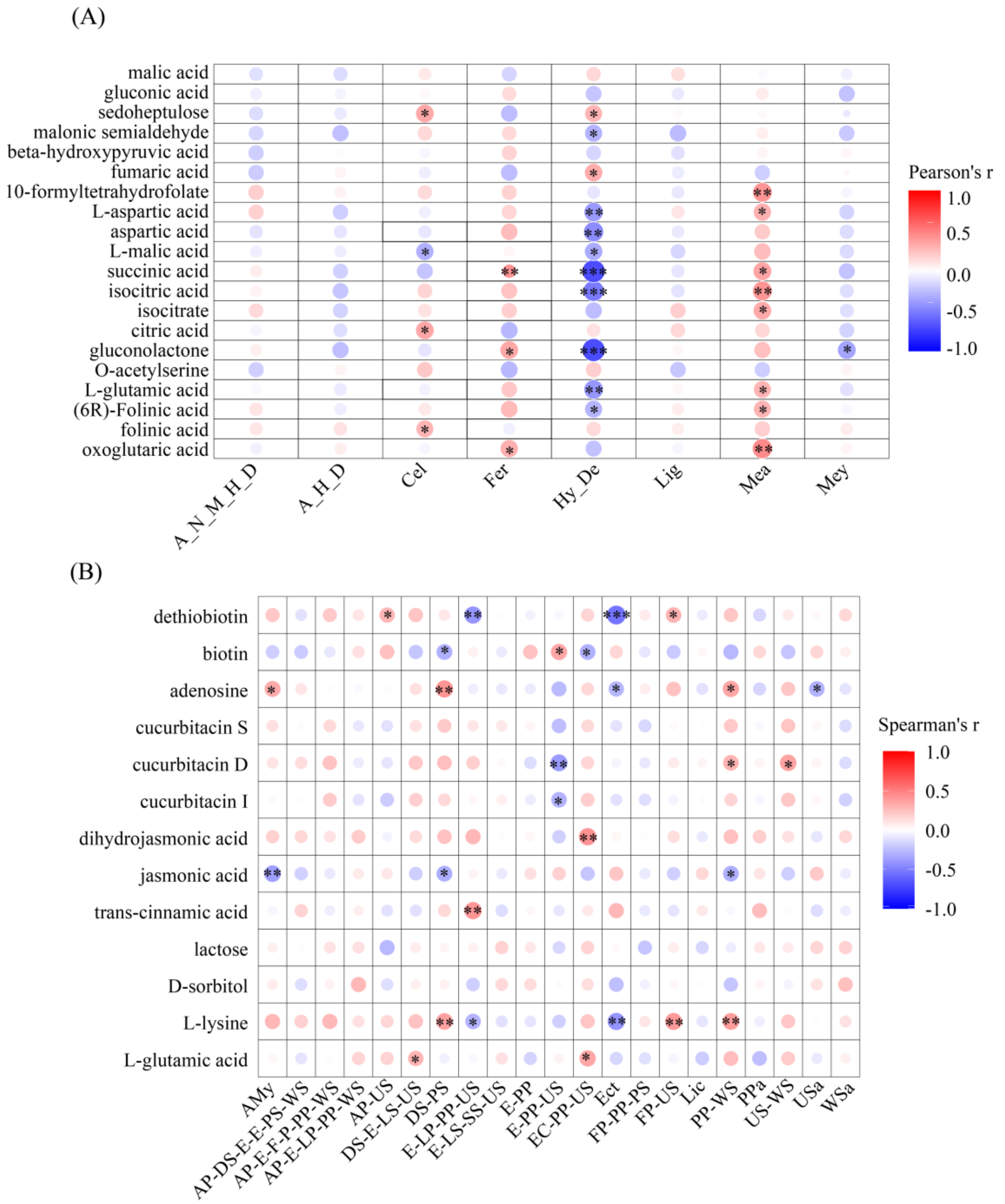

Fig. S3. Correlation Analysis Between Soil Metabolites and Predicted Microbial Functions. (**A**)bacteria, (**B**)fungi.

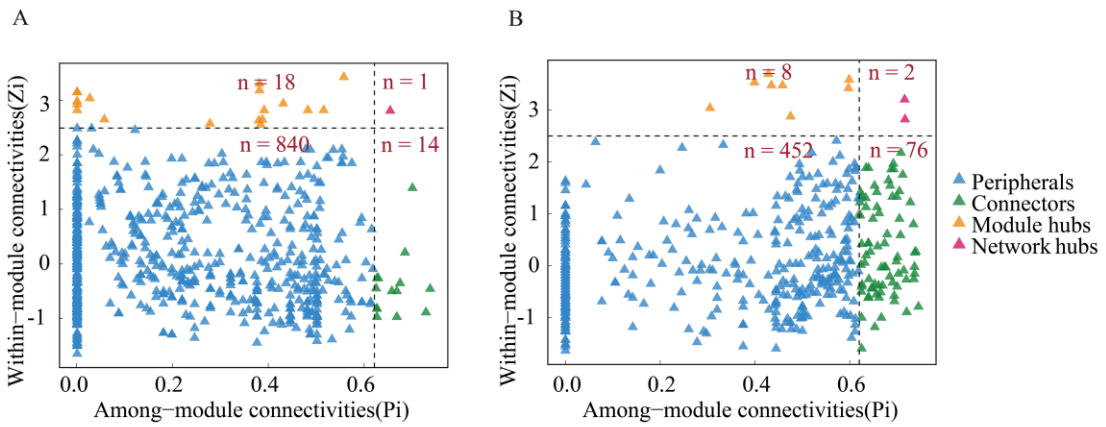

Fig. S4. Distribution of bacterial and fungal phylum based on their network roles: (**A**)bacteria, (**B**)fungi. Nodes in the network were classified as peripherals, modular hubs, or connectors.

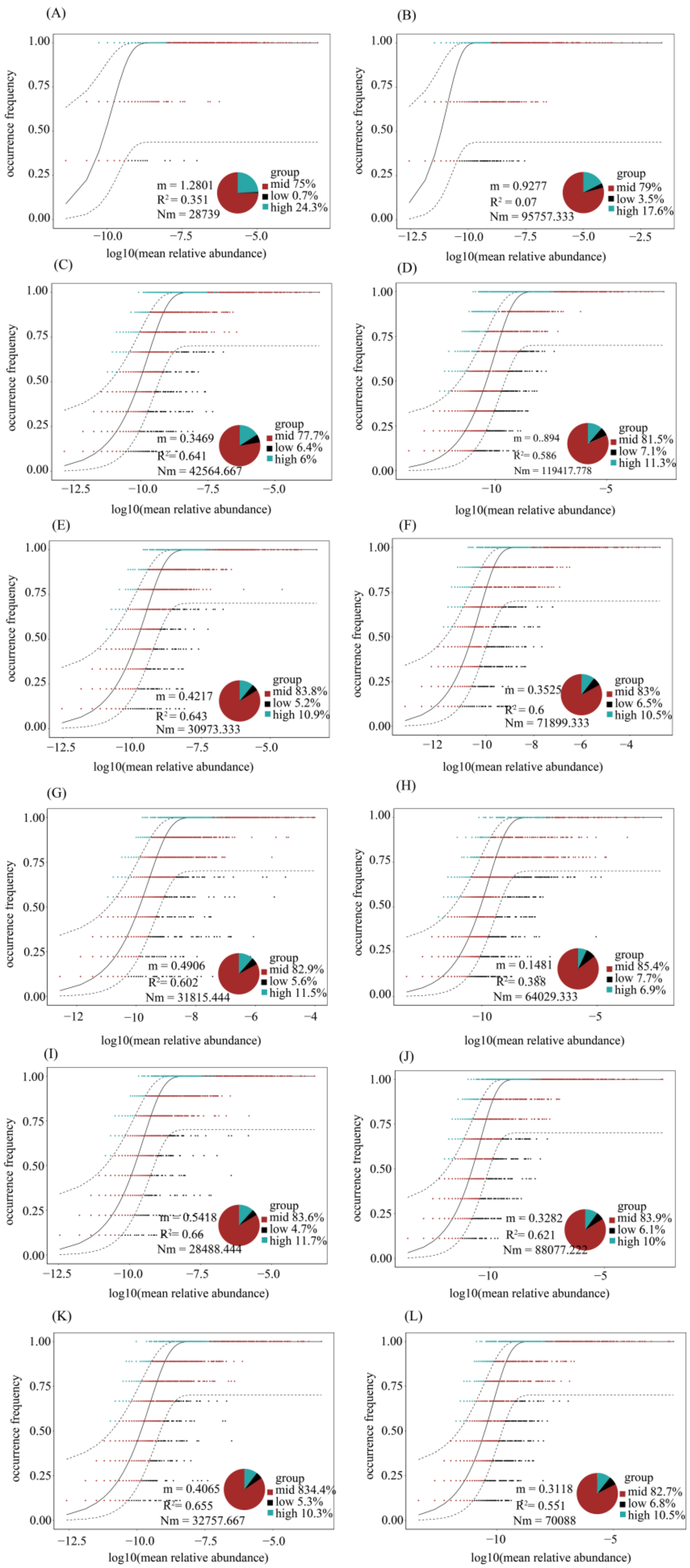

(A)
m = 1.2801
R² = 0.351
Nm = 28739
group
mid 75%
low 0.7%
high 24.3%
(B)
m = 0.9277
R² = 0.07
Nm = 95757.333
group
mid 79%
low 3.5%
high 17.6%
(C)
m = 0.3469
R² = 0.641
Nm = 42564.667
group
mid 77.7%
low 6.4%
high 6%
(D)
m = 0..894
R² = 0.586
Nm = 119417.778
group
mid 81.5%
low 7.1%
high 11.3%
(E)
m = 0.4217
R² = 0.643
Nm = 30973.333
group
mid 83.8%
low 5.2%
high 10.9%
(F)
m = 0.3525
R² = 0.6
Nm = 71899.333
group
mid 83%
low 6.5%
high 10.5%
(G)
m = 0.4906
R² = 0.602
Nm = 31815.444
group
mid 82.9%
low 5.6%
high 11.5%
(H)
m = 0.1481
R² = 0.388
Nm = 64029.333
group
mid 85.4%
low 7.7%
high 6.9%
(I)
m = 0.5418
R² = 0.66
Nm = 28488.444
group
mid 83.6%
low 4.7%
high 11.7%
(J)
m = 0.3282
R² = 0.621
Nm = 88077.222
group
mid 83.9%
low 6.1%
high 10%
(K)
m = 0.4065
R² = 0.655
Nm = 32757.667
group
mid 834.4%
low 5.3%
high 10.3%
(L)
m = 0.3118
R² = 0.551
Nm = 70088
group
mid 82.7%
low 6.8%
high 10.5%
occurrence frequency
log10(mean relative abundance)

Fig. S5. Fit of Sloan's neutral model for the analysis of community assembly processes. (**A**, **C**, **E**, **G**, **I**, **K**) bacteria, (**B**, **D**, **F**, **H**, **J**, **L**) fungi. The continuous black line represents the best-fitting neutral model; the dashed lines represent the 95% confidence intervals around the best-fitting neutral model m indicates the estimated migration rate, and R^2 indicates the fit to the neutral model. CK1: Natural forest; CK2: non-terraced sloped land; Y3-Y12: terraces aged 3-12 years.

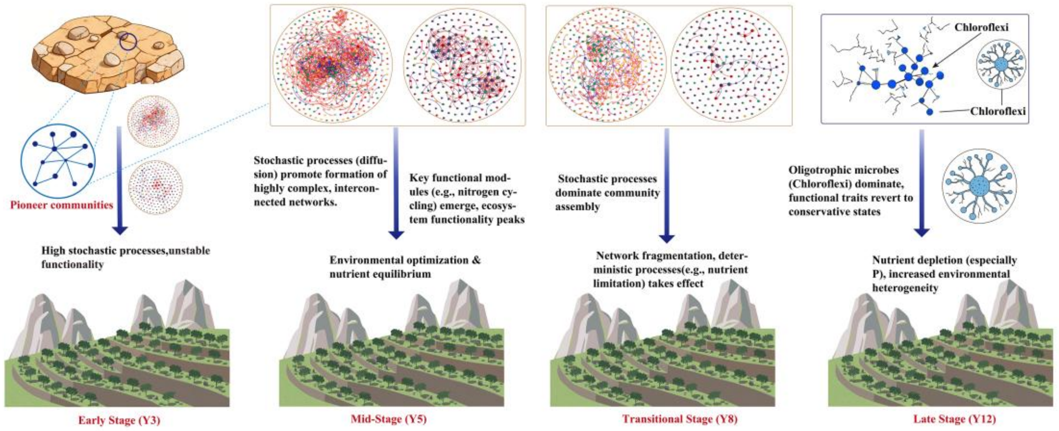

Fig. S6. Effects of terraces with different ages on soil microbial communities of citrus orchards.

Table S1

Physicochemical properties of citrus orchards terrace soils in different ages

	CK1	CK2	Y3	Y5	Y8	Y12
Clay	14.87 ±3.10	23.07±2.97	27.76±3.40	25.00±3.70	19.62±3.53	35.69±2.97
Sand	21.4±2.42	21.49±2.35	33.02±2.34	32.73±1.71	26.31±2.62	32.23±4.02
Silt	63.73±3.19	55.44±4.63	39.22±3.62	42.27±3.48	54.07±5.57	32.08±5.59
Rh	71.05±7.07	78.89±5.53	75.41±2.10	71.61±6.28	51.01±5.24	64.05±4.23
pH	3.98±0.22	3.83±0.30	4.64±0.61	4.4±0.33	5.61±0.75	4.27±0.27
OM	7.04±0.16	3.41±0.47	6.23±1.04	4.47±1.33	3.26±1.05	4.54±0.99
C/N	21.64±0.18	11.84±0.53	19.76±3.00	20.18±1.97	17.63±1.22	14.37±1.17
N/P	6.35±0.73	4.56±0.68	1.85±0.65	2.06±0.70c	1.38±0.37	2.98±1.58
C/P	137.57±17.05	54.09±8.65	35.47±9.23	41.39±13.25	24.19±6.06	46.00±27.64
AP	3.35±0.65	85.66±29.33	67.53±16.27	40.68±9.85	53.03±17.09	79.52±13.47
HN	238.00±48.50	133.78±15.43	192.89±16.53	146.22±21.08	133.78±34.52	205.33±19.34
AK	43.23±1.98	129.18±13.57	264.25±39.88	215.37±20.51	191.24±35.21	180.73±33.71
TK	8.52±0.89	8.36±0.57	11.11±1.15	10.01±0.64	12.17±0.77	16.76±0.97
ACa	0.67±0.00	0.71±0.03	0.79±0.25	0.83±0.10	0.84±0.11	0.77±0.06
ANa	1.13±0.10	1.25±0.03	1.44±0.24	1.77±0.19	1.72±0.20	2.02±0.12
AMg	1.13±0.10	1.25±0.04	1.33±0.05	1.39±0.04	1.43±0.09	1.48±0.03

CK1: Natural forest; CK2: non-terraced sloped land; Y3-Y12: terraces aged 3-12 years.

Table S2

Mantel test results of soil physicochemical properties and microbial communities

	Index	Soil Properties	r	*p*
**Bacteria**	**Abundance**	AP	0.157	0.041
		Clay	0.144	0.018
		OM	0.150	0.004
	**Diversity**	C/P	0.228	0.029
		N/P	0.097	0.039
		HN	0.138	0.045
		Silt	0.163	0.010
	**Composition**	C/P	0.272	0.002
		AP	0.216	0.012
		AK	0.182	0.024
**Fungi**	**Abundance**	C/N	0.206	0.004
	**Diversity**	N/P	0.121	0.030
		C/P	0.149	0.040
		AP	0.199	0.038

Table S3

Topological characteristics of symbiotic network of bacteria and fungi in terraced soil of citrus orchards of different years

		Y3	Y5	Y8	Y12
Bacteria	**Number of nodes**	319	368	333	300
	**Number of edges**	738	1387	700	550
	**Positive (%)**	86.18	85.15	87	88.36
	**Negative (%)**	13.82	14.85	13	11.64
	**Average degree**	4.602	7.431	4.192	3.66
	**Modularity**	0.667	0.626	0.786	0.879
	**Average clustering coefficient**	0.754	0.696	0.767	0.779
Fungi	**Number of nodes**	220	227	205	209
	**Number of edges**	302	521	244	279
	**Positive (%)**	95.7	91.36	95.9	92.83
	**Negative (%)**	4.3	8.64	4.1	7.17
	**Average degree**	2.727	4.581	2.38	2.67
	**Modularity**	0.906	0.752	0.92	0.922
	**Average clustering coefficient**	0.739	0.747	0.67	0.762

Y3-Y12: terraces aged 3-12 years.
